# Non-B DNA at the Interface of Metabolism, Genome Stability, and Inflammation

**DOI:** 10.3390/genes17101301

**Published:** 2026-10-19

**Authors:** Georgios Megalovasilis, Eleftherios Bochalis, Dionysios V. Chartoumpekis, Karen M. Vasquez, Ilias Georgakopoulos-Soares

**Affiliations:** 1 Division of Pharmacology and Toxicology, College of Pharmacy, Dell Pediatric Research Institute, The University of Texas at Austin, Austin, TX 78723, USA; georgios.megalovasilis@austin.utexas.edu (G.M.); eleftherios.bochalis@austin.utexas.edu (E.B.); 2 Department of Internal Medicine, Division of Endocrinology, Medical School, University of Patras, 26335 Patras, Greece; dchart@upatras.gr

**Keywords:** non-B DNA, metabolic disease, G-quadruplex, i-motif, R-loop, Z-DNA, inflammation, genomic instability, mitochondrial dysfunction

## Abstract

DNA most commonly adopts the canonical B-form double helix but can also form alternative conformations, collectively known as non-B DNA structures. These structures, including G-quadruplexes, i-motifs, hairpins, cruciforms, Z-DNA, H-DNA, R-loops, and other repeat-associated structures, have important roles in gene regulation, genome stability, and disease etiology. Emerging evidence suggests that non-B DNA is also involved in metabolic regulation and associated disorders. Here, we discuss how non-B DNA influences the expression of genes involved in insulin regulation, glucose and lipid metabolism, and other metabolic pathways. We also highlight their broader effects on genomic instability, mitochondrial dysfunction, and inflammatory signaling in diabetes, metabolic liver disease, obesity, and related disorders. Importantly, this relationship appears bidirectional, as metabolic and oxidative stress can alter the formation, processing, and stability of non-B DNA structures. Together, these findings position non-B DNA as a potential interface between metabolic stress, gene regulation, and tissue dysfunction.

## 1. Introduction

**Figure 1:**
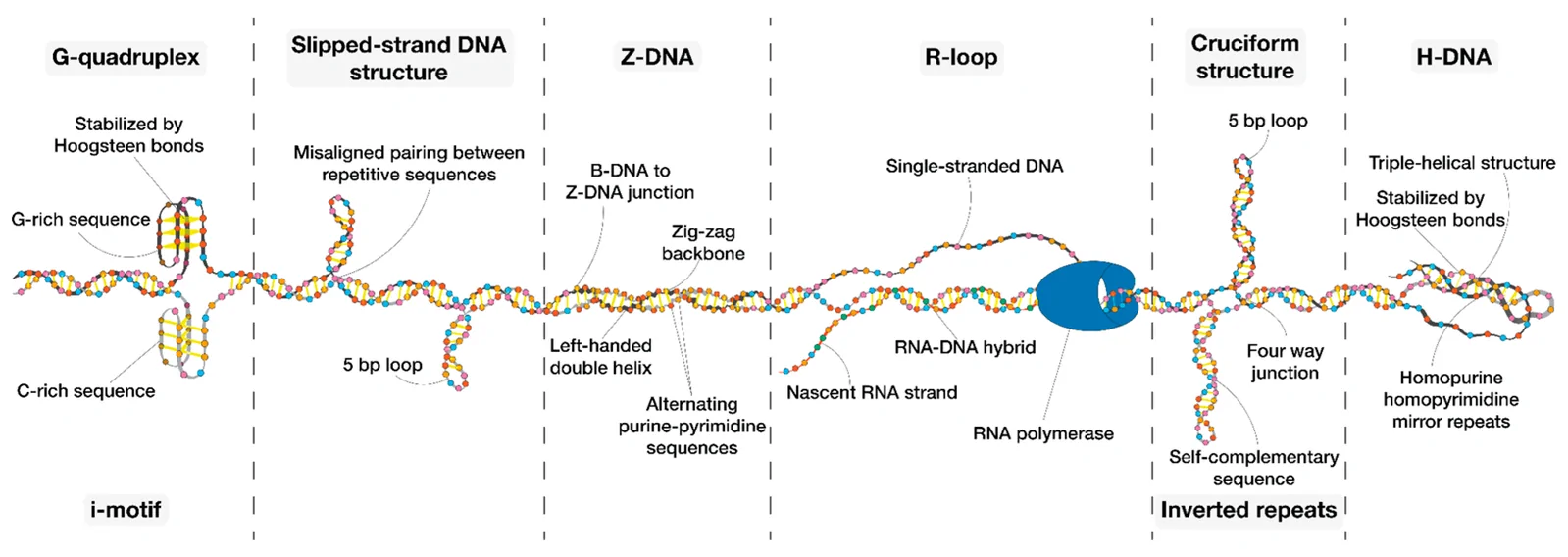
Schematic representation of non-B DNA structures.

Under physiological conditions, genomic DNA predominantly adopts the canonical right-handed B-form double helix [1]. DNA is, nevertheless, structurally dynamic, and certain repetitive sequences can diverge from the canonical conformation when the local physical and biochemical environment favors it [2,3,4]. These alternative conformations, broadly termed non-B DNA, include G-quadruplexes (G4s), i-motifs, Z-DNA, hairpins and cruciforms, slipped-strand DNA, H-DNA, and R-loops [5,6]. Their structural characteristics are illustrated in Figure 1.

G4s are four-stranded structures formed by guanine-rich sequences, featuring stacked planar G-quartets stabilized by Hoogsteen hydrogen bonds and monovalent cations [7]. In contrast, i-motifs form at cytosine-rich sequences through intercalated cytosine-cytosine (C-C^+^) base pairs [8]. Z-DNA adopts a left-handed double helix with a zigzag sugar–phosphate backbone, favored by alternating purine–pyrimidine sequences [9]. Hairpins arise from self-complementary sequences that fold into stem–loop structures, whereas cruciforms form at inverted repeats through the extrusion of two opposing hairpins [10,11]. Slipped-strand DNA structures result from misaligned pairing between repetitive sequences, producing extrahelical loops [12]. H-DNA forms at homopurine–homopyrimidine mirror repeats through intramolecular triple-helix formation, involving Hoogsteen or reverse Hoogsteen hydrogen bonding [13]. Finally, R-loops comprise an RNA-DNA hybrid and a displaced single-stranded DNA region, typically formed when nascent RNA hybridizes with the template DNA strand during transcription [14]. Non-B DNA structure formation is, therefore, promoted by the underlying sequence, but this alone does not establish that a non-B DNA structure will exist at a given locus at a particular time in a cell [15].

Several parameters of the cellular environment can drive a non-B-prone sequence to transition from the B-DNA to the non-B DNA state. A major contributing factor is the ionic environment within a cell, as cations modulate electrostatic repulsion within the DNA backbone, with different ions exerting distinct effects on structural stability [16]. Because the cellular environment contains a high concentration of biomolecules in addition to water and ions (macromolecular crowding), these molecular crowders have also been shown to play a role in determining non-B DNA structure and stability [17]. Chromatin state is another major factor. As non-B DNA structures are facilitated by negative supercoiling, processes such as replication, transcription, and DNA repair can promote their formation [18,19,20].

Non-B DNA structures have been implicated in a myriad of cellular functions, such as transcriptional regulation, DNA replication, genome organization, and recombination, among other biological processes [21,22,23,24,25]. Despite their important regulatory functions, non-B DNA-forming sequences can act as endogenous mutational hotspots [26,27,28,29,30,31]. Because non-B DNA structures can expose single-stranded regions and contain sequence contexts susceptible to DNA damage, they can act as hotspots for DNA damage and mutagenesis. For example, guanine-rich sequences are particularly susceptible to oxidative damage, including 8-oxo-guanine formation [32], while cytidines exposed within DNA stem–loop structures can be preferentially targeted by APOBEC3A, generating recurrent mutation hotspots [30]. Once damaged, these structures can also interfere with DNA repair, allowing lesions to persist. Unresolved lesions can lead to DNA double-strand breaks and promote mutations, translocations, copy number alterations, and repeat expansions [27]. Non-B DNA-induced genetic instability can result in functional consequences and has been associated with disease development, inflammation, and aging [31,33,34,35].

The relevance of these structures to metabolic disease is becoming increasingly recognized. G4s and i-motifs have been linked to the regulation of insulin and other metabolic genes, while sequence variation within these regions can alter their formation dynamics and their regulatory effects [36]. Other non-B DNA structures have also been implicated in metabolic disease through distinct mechanisms. For example, a Z-DNA-forming region in the *SLC11A1* promoter provides a possible connection between structural regulation of an immune-related gene and type 1 diabetes susceptibility [37], while abnormal R-loop accumulation in the liver has been associated with greater disease severity in fatty liver disease [38]. These represent examples from a growing body of studies linking non-B DNA structures to metabolic disease. Emerging evidence suggests that their effects extend from the regulation of individual metabolic genes to broader processes such as genomic instability and inflammatory signaling. This review examines these emerging roles and the mechanisms through which non-B DNA may contribute to the development and progression of metabolic disease.

## 2. Non-B DNA in Insulin Gene Regulation

**Figure 2:**
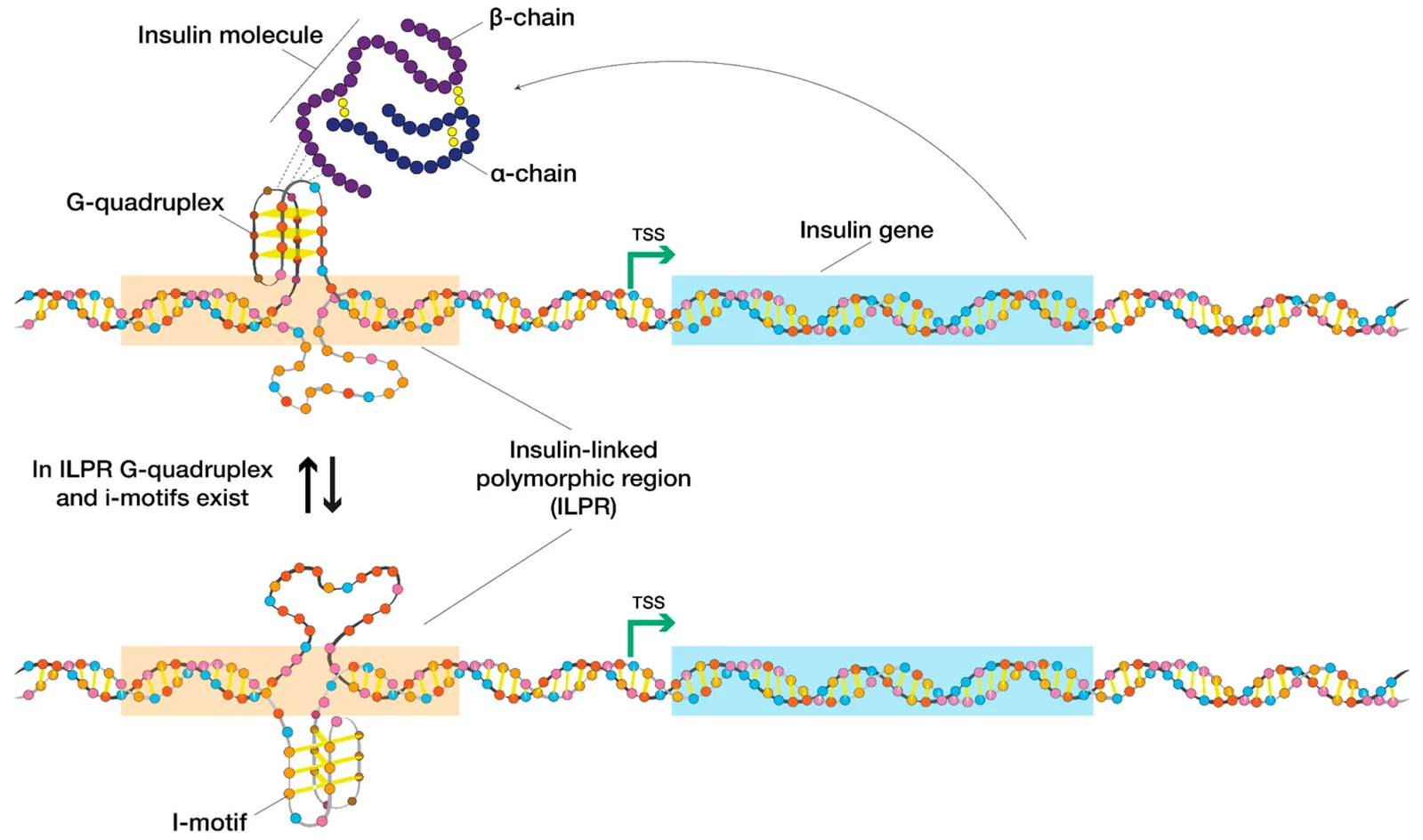
Insulin expression is regulated via mutually exclusive G-quadruplexes and i-motif structures formed in the insulin-linked polymorphic region. Upstream of the *INS* gene transcription start site (green arrow) lies the ILPR region, a tandemly repeated array with the propensity to form G-quadruplexes or i-motifs. (**Top**) Insulin itself can bind to G-quadruplexes formed on the G-rich strand. This interaction is mediated mainly between insulin’s β-chain and the loop backbone of the G-quadruplex. (**Bottom**) The complementary C-rich strand can form i-motif structures, which are proposed to fine-tune insulin expression.

Insulin is a central regulator of metabolic homeostasis, and alterations in its synthesis, secretion, or signaling can contribute to the development and progression of several metabolic disorders, including, but not limited to, diabetes [39]. After the HLA chromosome region, the insulin gene locus represents the second strongest genetic association linked to type 1 diabetes [40]. ILPR is a GC-rich variable tandem repeat upstream of the insulin gene [41]. ILPR provides an example of how non-B DNA can affect metabolic gene regulation (Figure 2). The complementary strands of the ILPR have been shown to adopt non-B DNA conformations [42]. Specifically, the G-rich strand favors G4s, while the C-rich strand promotes i-motif formation [43]. Earlier studies suggested a simple relationship between G4 formation and *INS* promoter activity, as transcriptionally active ILPR variants showed a greater propensity to form G4 structures [41,44] (Figure 2). Subsequent analyses, however, indicated that G4 formation alone cannot explain transcriptional differences. Multiple ILPR variants with different reported transcriptional activities were capable of forming G4s in vitro, arguing against a simple relationship between the presence of a G4 and promoter activity [45,46].

More recent work supports the idea that G4s and i-motifs are components of a dynamic regulatory system rather than independent structural switches (Figure 2). G4s have been suggested to assist in establishing a transcriptionally permissive state. In contrast, the complementary i-motif may fine-tune transcription, with its stability influencing insulin expression [47]. This interpretation is consistent with experiments showing that G4 and i-motif structures are mutually exclusive within duplex ILPR DNA. As a result, they compete for formation rather than coexisting on opposite strands [43] (Figure 2). The C-rich strand can also form a partially folded, likely triplex, intermediate. This suggests that the ILPR can adopt a variety of structures beyond simply switching between G4s and i-motifs [42]. Both the i-motif and these intermediate structures are mechanically stable enough to resist forces generated by RNA polymerase. They may therefore influence insulin expression by directly affecting transcriptional progression, in addition to recruiting regulatory proteins [43].

One unusual feature of the ILPR system is that insulin itself can recognize the G4 formed within its own regulatory region [48]. ILPR G4s selectively capture insulin, and this interaction is primarily mediated by the insulin β-chain [49] (Figure 2). High-resolution structural experiments have further localized insulin binding to the sugar–phosphate backbone of ILPR G4 loops rather than to the guanine tetrads themselves [50]. This emphasizes that loop architecture is a contributing factor for protein recognition. These reports suggest that insulin may influence the regulation of its own gene by binding ILPR G4s, although the nature of this interaction in vivo remains unclear.

Non-B DNA regulation of *INS* also extends beyond ILPR. G4-forming sequences flank a splicing regulatory element in *INS* intron 1, where DNA and nascent RNA can form G4-like structures in vitro [51]. This study suggests that G4s could also influence *INS* co-transcriptional splicing. The same region contains the type 1 diabetes-associated rs689 variant, a major genetic risk factor for type 1 diabetes [40]. This may provide a link between local G4 formation, insulin RNA processing, and disease-associated variation [52].

Collectively, these studies show that non-B DNA structures may regulate *INS* at multiple levels, linking sequence and structural variation to both transcription and RNA processing. However, their physiological roles in pancreatic β cells remain to be fully established.

## 3. G-Quadruplexes in Metabolic Gene Regulation and Disease

Beyond the *INS* locus, studies of individual G4-forming regions have linked these structures to the regulation of several major metabolic pathways, including glucose and lipid metabolism, adipocyte differentiation, thermogenesis, and cholesterol homeostasis [53,54,55,56,57]. Their effects on transcription, however, are not uniform, as G4 formation can either promote or repress gene expression. The specific effect of a G4 depends on different factors, including underlying sequence composition, genomic context, and regulatory environment, such as local methylation status. This context-dependent role is illustrated by the impact of G4-forming regions in the *GCK* gene locus. GCK is a key enzyme in glucose metabolism and homeostasis, and mutations in the *GCK* gene have been associated with monogenic diabetes MODY 2 in cases of loss of function and with hypoglycemia in cases of gain of function, as glucose sensing is affected [58]. At this locus, methylation has been shown to destabilize G4 formation and result in suppression of the *GCK* promoter and enhancer regions. On the other hand, demethylation restores the potential for G4 formation and is associated with greater regulatory activity [53]. This methylation-dependent role of G4s in metabolic gene regulation has also been described for *TM6SF2* [59], which regulates lipid handling and is associated with fatty liver disease, as its loss of function enhances triglyceride accumulation in the liver [60]. These studies suggest that epigenetic changes in metabolic disease may influence gene expression partly by altering the non-B DNA structures present at regulatory regions.

In addition to the methylation-dependent effects, G4s can directly regulate the expression of genes involved in lipid metabolism and energy homeostasis. *ACBP* participates in lipid metabolism, and its dysregulation has been associated with metabolic and liver disease [61]. Its promoter G4 has been reported to recruit G4-binding proteins and promote transcription, while disruption of this structure can reduce *ACBP* expression and lipid accumulation [54]. Similarly, a G4-forming sequence in the *ABCA1* promoter may promote *ABCA1* expression, possibly through NONO recruitment. Deleting this G4-forming sequence increased cholesterol accumulation in macrophages [55]. This is metabolically relevant because *ABCA1* regulates cholesterol homeostasis and has been linked to obesity, insulin resistance, and type 2 diabetes [62]. In contrast, G4-forming regions in *UCP1*, which supports brown-fat thermogenesis [63], and *ACC1*, a gene involved in fatty acid synthesis [64], were reported to repress transcription [56,65]. G4-mediated repression has also been reported for *CES2*, an enzyme that hydrolyzes triglycerides, diglycerides, and monoglycerides [66]. Two G4-forming regions were identified upstream of *CES2,* and treatment with the G4-stabilizing compound pyridostatin reduced *CES2* expression and activity and increased triglyceride accumulation in hepatocytes [67]. However, the compound also reduced the expression and promoter binding of *HNF4α*, a transcriptional activator of *CES2*, so these effects cannot be attributed solely to the *CES2* G4s. Beyond individual metabolic genes, increased G4 detection has been reported during adipocyte differentiation. Treatment with the G4-stabilizing ligand Braco-19 during differentiation reduced lipid accumulation and expression of the adipogenic markers PPARG, AP2, and LEP [57]. These findings suggest a role for G4 regulation in adipogenesis, although the underlying mechanism remains unclear.

Another aspect of the relationship between G4s and metabolic disease involves genetic variation within potential G4-forming sequences. Thousands of metabolic disease-associated variants occur within G4-forming sequences, with disease-associated alleles generally predicted to form less stable G4s. These variants may also alter transcription-factor binding, including binding sites for *PDX1* [68], a key regulator of pancreatic β-cell function and insulin expression [69]. Similarly, variants in *MICB* and *PLA2G6* (both involved in metabolic disease, affecting directly and indirectly immuno-metabolic and lipid handling pathways) can alter G4 stability and are associated with differences in expression levels [70]. These findings suggest that genetic variants within G4-forming regions can influence gene regulation by altering both G4 structure and interactions with regulatory proteins.

In conclusion, G4s can both promote and repress transcription under different conditions, are impacted by epigenetic alterations, and influence the effects of genetic variation within metabolism-related genes. Through these diverse mechanisms, G4s can affect pathways involved in glucose and lipid metabolism, cholesterol homeostasis, thermogenesis, and adipocyte function, providing multiple links between G4 formation and metabolic disease.

## 4. I-Motifs in Metabolic Gene Regulation and Tissue Injury

Beyond insulin regulation, i-motifs have also been linked to other genes involved in lipid metabolism and metabolic disease. One example is *BCL2*, which encodes an anti-apoptotic protein [71]. The i-motif-binding compound A22 was shown to stabilize an i-motif in the *BCL2* promoter and increase its expression [72]. In fatty liver disease models, A22 also reduced hepatocyte apoptosis, liver injury, inflammation, and fibrosis, while improving glucose tolerance [72]. This study suggests that targeting the i-motif of the *BCL2* promoter may protect against metabolic liver injury. However, the effects of A22 were not shown to depend specifically on the endogenous *BCL2* i-motif.

A different effect has been reported for *ACC1*. C-rich sequences in the *ACC1* promoters can form i-motifs, and reporter experiments indicated that these regions repress transcription [73]. This differs from the increased *BCL2* expression observed after A22 treatment and illustrates the context-dependent effects of non-B DNA on gene regulation. However, *ACC1* regions also contain complementary G4-forming sequences, making it difficult to determine the individual contribution of each non-B DNA structure.

These studies suggest that i-motifs can influence genes involved in lipid metabolism and cellular responses to metabolic stress. Depending on the locus and regulatory context, their formation may promote or repress gene expression. However, direct evidence linking specific endogenous i-motifs to metabolic disease remains limited.

## 5. R-Loops in Metabolic Disease and Inflammation

R-loops are three-stranded structures consisting of an RNA-DNA hybrid and a displaced single strand of DNA [74]. They form mainly during transcription and have roles in gene expression and DNA repair, but when they persist, they can become a source of replication stress and DNA damage [75,76]. DNA damage is itself broadly linked to inflammation [77], and R-loops connect to it in two ways. Persistent R-loops can generate cytoplasmic RNA-DNA hybrids that are sensed by cGAS and activate innate immune signaling [78]. This has been demonstrated in cells lacking R-loop-resolving helicases, where excess R-loops trigger inflammatory responses through cGAS-STING signaling directly or through micronuclei [79,80]. R-loops can also impact transcription itself and alter the expression of metabolic and inflammatory genes [81]. Both events have been described in metabolic tissues, although the direction of the effect is inconsistent, with some studies attributing pathology to R-loop accumulation and others to R-loop loss.

**Figure 3:**
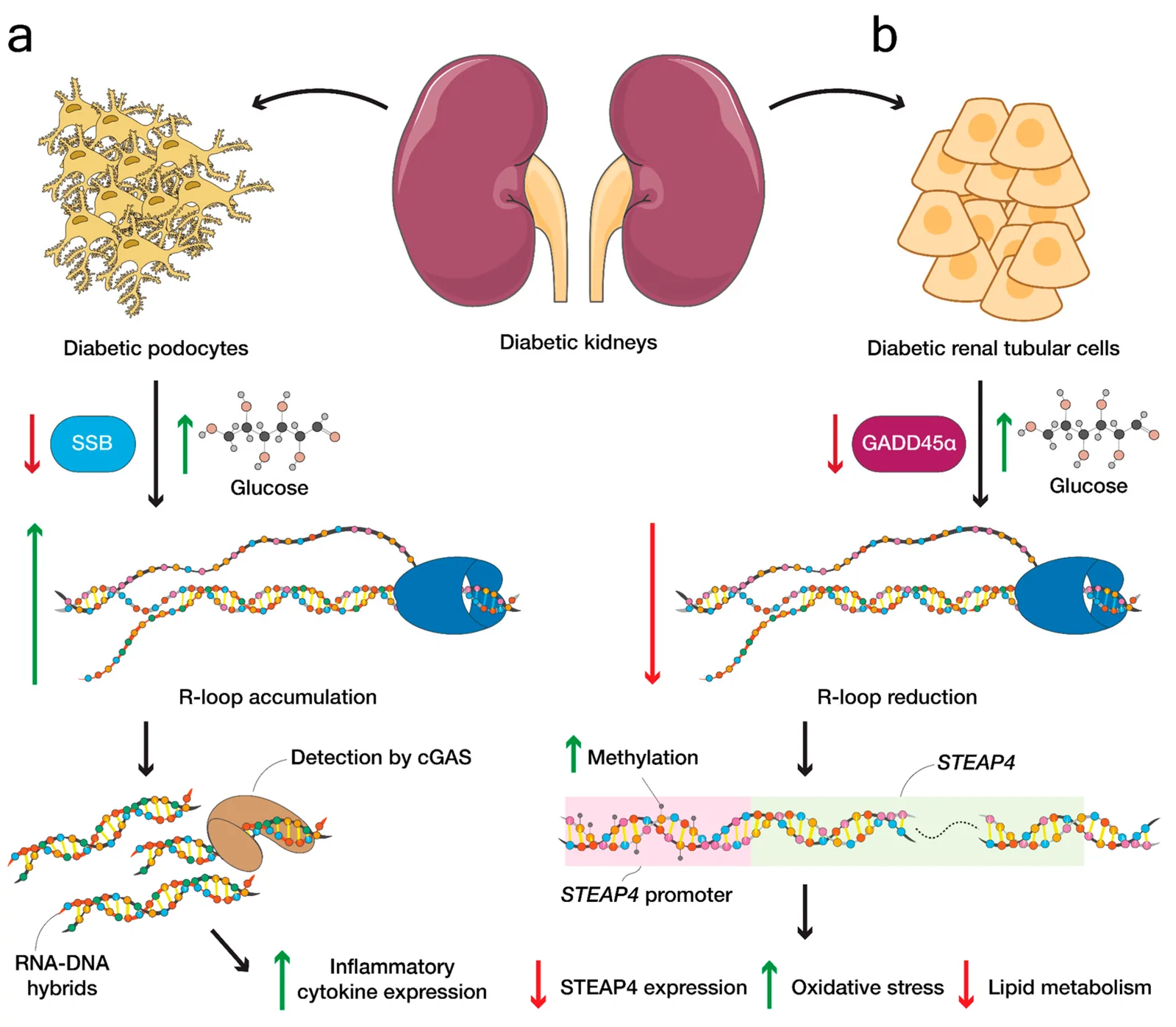
Opposing R-loop changes are observed in diabetic kidneys: (**a**) In diabetic podocytes, high glucose decreases *SSB* expression and promotes R-loop accumulation, resulting in cGAS detection of RNA-DNA hybrids. This activates the cGAS-STING pathway, leading to increased inflammatory cytokine expression. (**b**) In diabetic renal tubular cells, high glucose decreases *GADD45a* expression and reduces R-loop formation at the *STEAP4* promoter. *STEAP4* expression is suppressed, with impaired lipid metabolism and increased oxidative stress also observed.

The kidney illustrates this context dependency well (Figure 3). Diabetic kidney disease is now viewed as a chronic inflammatory condition rather than a purely metabolic one [82], with podocyte injury central to its progression [83]. In diabetic podocytes, high glucose reduced the expression of the R-loop regulator SSB and promoted R-loop accumulation, which in turn increased inflammatory cytokine expression through cGAS [84] (Figure 3a). In renal tubular cells, the opposite was reported. High glucose was shown to reduce R-loop formation at the *STEAP4* promoter through downregulation of GADD45α [85]. This increased promoter methylation and suppressed *STEAP4*, a regulator of glucose metabolism, mitochondrial function, and insulin resistance [81,86] (Figure 3b). Reduced *STEAP4* expression was accompanied by impaired lipid metabolism, mitochondrial dysfunction, and oxidative stress. Since *STEAP4* deficiency can also promote inflammation [87], loss of these R-loops may contribute indirectly to inflammatory signaling, although this was not directly tested. These studies propose that both excessive R-loop accumulation and/or R-loop loss can be harmful, depending on genomic location and regulatory function.

A similar loss of R-loops has been linked to impaired diabetic wound healing. The long noncoding RNA DSP-AS1 can form R-loops at the promoter of *DSP*, which encodes the cell-adhesion protein desmoplakin [88]. Reduced R-loop formation was reported to limit promoter demethylation and *DSP* expression, impairing restoration of the epithelial barrier [89]. This suggests that loss of regulatory R-loops can contribute to defective tissue repair in diabetes, although the structure-specific evidence to date has only been shown in cultured cells.

In the liver, by contrast, R-loop accumulation has been linked to metabolic tissue injury. Hepatocyte-specific loss of the splicing factor SRSF1 increased R-loops and DNA damage, followed by steatosis, hepatocyte death, inflammation, and fibrosis, producing a NASH-like phenotype [90]. R-loops and DNA damage appeared before overt liver injury, suggesting an early contribution to disease development. A similar connection involves the splicing factor SRSF3, which is degraded during MAFLD and MASH [91]. Its loss increased R-loops and DNA damage, while stabilizing SRSF3 in MASH mice reduced both R-loops and DNA damage [38]. While these studies associate impaired RNA processing with R-loop accumulation and liver damage, neither of them directly removed R-loops in vivo. Such experiments might better determine their contribution to liver inflammation.

R-loops can also support cellular adaptation to metabolic stress, although this may favor tumor growth. In MASH-related hepatocellular carcinoma, lipid excess led to the accumulation of NONO at DNA breaks [92]. R-loops, formed at these sites, altered local genome organization and increased expression of genes involved in proliferation, lipid metabolism, and DNA repair. Removing these R-loops reduced tumor-cell survival under lipid overload [92]. More broadly, dysregulated R-loop formation has been implicated in hepatocellular carcinoma through its effects on genomic instability, DNA repair, and transcriptional regulation [93]. The role of R-loops in inflammatory gene expression is less clear. Across different models, including atherosclerosis, NASH, and gout, strongly upregulated genes overlapped with fewer annotated R-loops than downregulated genes [94]. The authors proposed that reduced R-loop persistence may facilitate inflammatory gene expression, but the analysis used reference annotations rather than R-loop measurements from the same samples. Other studies have linked R-loop regulators to inflammatory gene expression [95] and excess R-loops to inflammation through cell death [96]. Nevertheless, these findings do not clearly establish metabolic roles.

In summary, R-loops connect metabolic disease to both tissue injury and gene regulation. Their accumulation can promote DNA damage and inflammatory signaling, while loss of R-loops in specific promoters can impair metabolic gene expression or tissue repair. Their effects seem to depend on their location and processing rather than simply on their overall abundance. While these experiments are compelling, further studies are required to demonstrate structure formation in vivo.

## 6. Mitochondrial Non-B DNA and Metabolic Dysfunction

**Figure 4:**
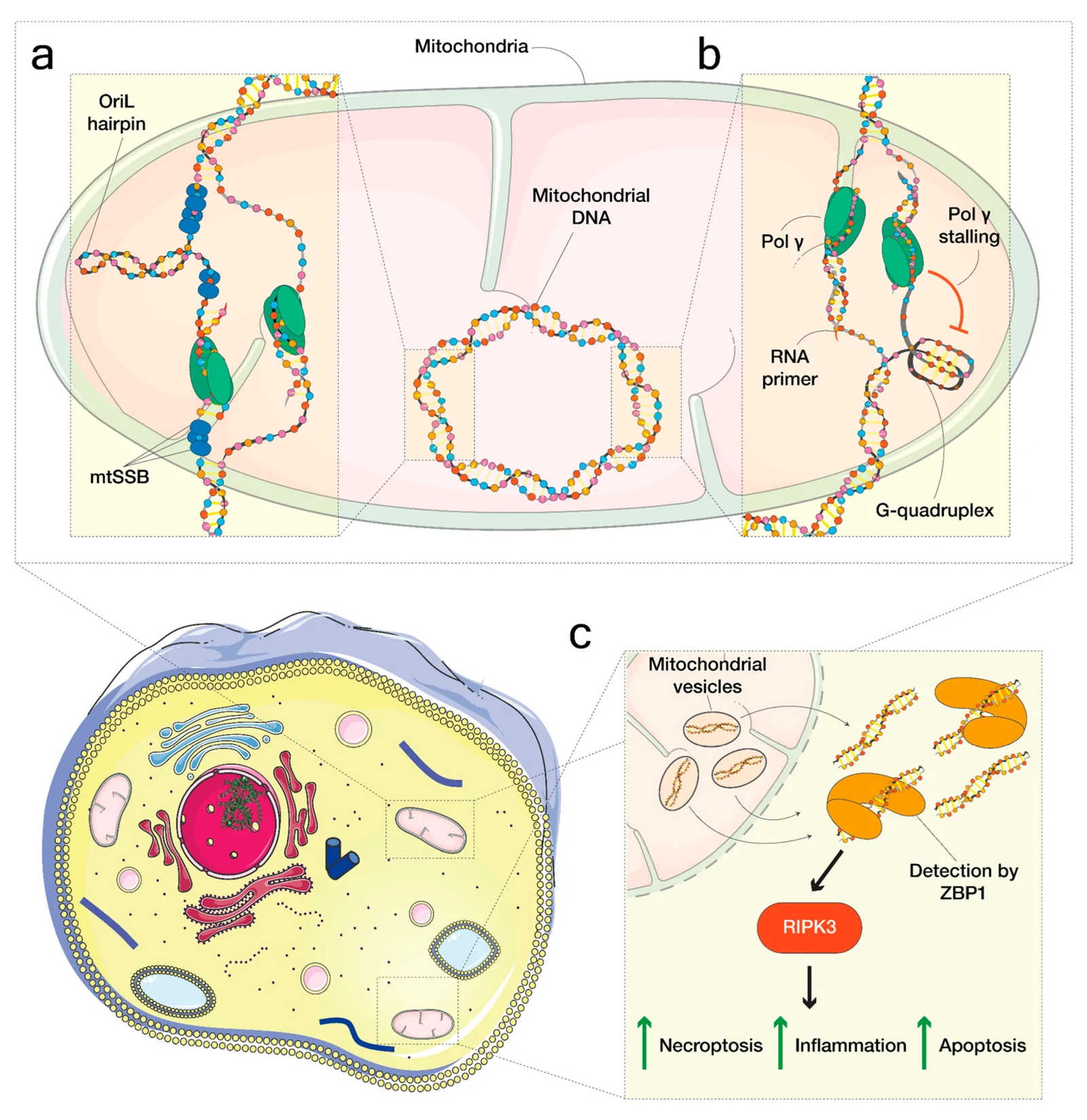
Non-B DNA structures affect both mitochondrial DNA maintenance and inflammation signaling: (**a**) During strand displacement replication, mtSSB coats the displaced parental heavy strand. At OriL, hairpin formation limits mtSSB binding, leaving the loop accessible to POLRMT for RNA primer synthesis and initiation of new light-strand DNA synthesis. (**b**) G-quadruplex formation on the template strand can lead to Pol γ stalling if left unresolved. (**c**) Stressed mitochondria release mtDNA in the Z-DNA conformation in the cytoplasm. This is detected by ZBP1, which engages RIPK3 to induce necroptosis, inflammation, and apoptosis.

Cells need to ensure the integrity and expression of their mtDNA to support energy metabolism, and non-B DNA can influence these processes [97]. At the origin of light-strand replication, the exposed parental heavy strand forms a hairpin that supports replication initiation [98,99]. This hairpin structure limits the binding of mtSSB to the mtDNA, allowing the POLRMT polymerase to synthesize the primer needed for new light-strand synthesis [98,100] (Figure 4a). While the OriL hairpin is important for mtDNA replication, hairpin-forming regions elsewhere in mtDNA have been shown to contribute to genome instability [97]. In the same study, deletion breakpoints were frequently found within or near predicted hairpins, including some surrounding OriL and near the D-loop.

A more direct connection of non-B DNA to metabolic liver disease, in the context of mitochondrial function, involves endonuclease G. EndoG is a protein implicated in apoptosis, mitochondrial genome integrity, and autophagy [101]. Its activity has been associated with obesity and various liver pathophysiologies. Specifically, *EndoG* expression was reduced in the liver of patients with MASLD, while its deficiency promoted hepatic fat accumulation in male mice, even in the absence of obesity [102]. A study reported that EndoG loss increased mitochondrial RNA-DNA hybrids and impaired protein synthesis and respiration [103]. Similarly, *RNASEH1* (another regulator of mitochondrial RNA-DNA hybrids) mutations were linked to mtDNA instability and respiratory dysfunction [94]. However, both enzymes have additional roles in RNA processing or DNA replication, so these defects cannot be attributed solely to the RNA-DNA hybrids.

G4s can similarly interfere with mitochondrial genome function (Figure 4b). G4-forming sequences have been associated with mtDNA deletions and replication stalling [104,105,106,107]. Pharmacological perturbation of mitochondrial G4s was shown to impair mitochondrial transcription and respiratory function [108]. This suggests that mitochondrial G4s can affect cellular energy metabolism by disrupting mtDNA transcription and/or replication (Figure 4b). A disease-associated example comes from the Leigh syndrome variant m.10191T>C, which increases local G4 formation, interferes with mtDNA replication, and causes a severe neurodegenerative disorder [109]. Targeting G4s in patient-derived cells reduced the relative abundance of the mutant mitochondrial genome and improved respiratory function. This supports the connection among disease-associated genetic variation, mitochondrial G4 formation, and energy metabolism.

In summary, mitochondrial RNA-DNA hybrids and G4s can affect mitochondrial respiration through changes in genome integrity and gene expression. These effects provide a potential association between non-B DNA and metabolic dysfunction, although direct evidence in the context of metabolic disease remains limited.

## 7. Z-DNA in Mitochondrial Dysfunction and Inflammatory Tissue Injury

Z-DNA has also been implicated in processes affecting mitochondrial function. In particular, emerging evidence links mitochondrial Z-DNA accumulation to inflammatory cell death. A study in diabetic kidney disease reported that stressed mitochondria accumulate and release DNA in the Z-conformation, which is then recognized by Z-DNA-binding protein 1 [110] (Figure 4c). ZBP1 activation might, then, promote proinflammatory signaling and necroptosis, a form of inflammatory cell death [111,112]. Z-DNA may, therefore, mediate the activation of inflammation and cell death in response to metabolic disturbances (Figure 4c).

ZBP1 expression was also found to be elevated in renal tissue from patients with diabetic nephropathy and correlated with tubular injury and impaired renal function [113]. Mechanistically, ZBP1 can interact with RIPK3 to promote necroptosis (Figure 4c). ZBP1 knockdown reduced necroptosis, inflammation, and fibrosis in diabetic mice and in tubular cells exposed to high glucose and advanced glycation end products [113]. These findings establish a functional contribution of ZBP1 to diabetic tubular injury, but the study did not determine whether Z-DNA was its activating ligand. This distinction is illustrated by steatotic liver ischemia/reperfusion injury. In this setting, ZBP1 promoted RIPK1-dependent apoptosis and inflammation, but deletion of its Z-nucleic acid-binding domains did not prevent injury. Instead, oxidative stress promoted ZBP1 aggregation and activation [114]. Thus, ZBP1 can contribute to injury in metabolically affected tissue without requiring Z-nucleic acid sensing. ZBP1 can also recognize endogenous Z-RNA, a left-handed form of double-stranded RNA, to activate inflammatory cell death [115]. Although RNA-only structures are not the focus of this review, Z-RNA is an alternative ZBP1 ligand that must be considered when attributing ZBP1-dependent injury to Z-DNA (see Section 9).

In an obesity- and hypertension-associated model of heart failure with preserved ejection fraction, mitochondrial dysfunction was associated with Z-DNA accumulation and ZBP1-dependent inflammatory cell death. Restoring NAD^+^ levels or suppressing ZBP1 reduced susceptibility to atrial fibrillation [116]. However, a contribution from Z-RNA was not excluded. These findings suggest that mitochondrial Z-DNA signaling may contribute to cardiometabolic complications as well.

## 8. Nuclear Z-DNA Regulation and Susceptibility to Type 1 Diabetes

A separate link between Z-DNA and metabolic disease comes from the regulation of genes involved in autoimmune diabetes. *SLC11A1* encodes a phagosomal ion transporter that influences immune-cell function [117]. Its relevance to diabetes is supported by experiments in non-obese diabetic mice, in which silencing of *SLC11A1* reduced diabetes frequency [118]. The human *SLC11A1* promoter contains a repeat capable of forming Z-DNA [119], providing a potential link between local DNA structure, gene expression, and disease susceptibility.

Different alleles of this repeat showed different effects on basal and stimulus-dependent transcription [120]. Later studies linked the region to HIF-1-dependent regulation and Z-DNA formation. During macrophage differentiation, recruitment of the SWI/SNF chromatin-remodeling complex promoted Z-DNA formation, while depletion of its BRG1 component reduced both Z-DNA formation and *SLC11A1* expression [119,121]. These findings support a regulatory role for Z-DNA in this region, although the experiments were not performed in diabetes models. Evidence directly connecting the region to type 1 diabetes is also limited. A large genetic study found the strongest disease association at a different, intronic *SLC11A1* variant, and the association was not reproduced in a family-based analysis [122]. Therefore, the promoter repeat and its ability to form Z-DNA have not yet been established as causal factors in type 1 diabetes.

Z-DNA may also influence central immune tolerance, which prevents immune responses against pancreatic β cells. In medullary thymic epithelial cells, promoter Z-DNA was linked to DNA breaks and increased chromatin accessibility at genes regulated by the autoimmune regulator AIRE. These changes helped prepare promoters for AIRE-dependent expression of tissue-restricted antigens [123]. The importance of thymic antigen expression in diabetes has been demonstrated separately. Deleting thymic *Ins2* in *Ins1*-deficient mice caused insulin-directed autoimmunity and diabetes while preserving pancreatic *Ins2* expression [124]. Together, these studies suggest that Z-DNA-dependent promoter regulation could contribute to immune tolerance against pancreatic antigens. However, they do not show that Z-DNA directly regulates thymic insulin expression or affects diabetes susceptibility.

## 9. Z-Nucleic Acids in Innate Immunity and Inflammation

The ZBP1-dependent responses described above are part of a broader surveillance system for Z-nucleic acids. ZBP1 and the p150 isoform of ADAR1 are the only mammalian proteins with Zα domains, which can recognize Z-DNA and Z-RNA [125]. Upon binding, ZBP1 promotes RIPK3 signaling through its RHIM domains to trigger necroptosis and apoptosis [125,126] (Figure 4c). The most studied Zα ligands are of viral origin. For example, replicating influenza A virus generates nuclear Z-RNA that activates ZBP1 and kills infected cells [126,127]. Endogenous nucleic acids can also induce the same response. Specifically, ADAR1 binds and edits double-stranded RNA derived from endogenous retroelements, particularly inverted SINE and Alu repeats, and mutation of its Zα domain allows these transcripts to accumulate and activate ZBP1, causing type I interferon-driven autoinflammation in mice [128,129,130]. The corresponding ADAR1 mutations cause Aicardi-Goutières syndrome in humans [131]. Retroelement-derived Z-nucleic acids similarly trigger ZBP1-dependent necroptosis and inflammation in skin and intestine when RIPK1 function is lost [132,133]. Of particular relevance to the mitochondrial findings discussed above, doxorubicin-induced mitochondrial genome instability promotes Z-DNA formation, which ZBP1 stabilizes and presents to cGAS in a complex also containing RIPK1 and RIPK3, sustaining interferon signaling and driving cardiotoxicity [134]. These mechanisms connect Z-nucleic acid sensing to the cGAS-STING and necroptotic pathways already implicated in the metabolic tissues discussed here. On top of that, chronic low-grade inflammation is itself a defining feature of obesity and type 2 diabetes [135]. Whether endogenous Z-DNA or Z-RNA contributes to this inflammatory state, however, has not been directly tested. Z-nucleic acid sensing represents a broad and rapidly evolving field that extends beyond the scope of this review. For a more detailed discussion, we refer readers to dedicated reviews on this topic [125,136,137,138].

Regarding Z-DNA, the strongest evidence for its implication in metabolic disease relates to mitochondrial stress and ZBP1-dependent inflammatory injury. Nuclear Z-DNA provides a complementary regulatory perspective. While its contribution to autoimmune diabetes is suggested by separate studies, its specific effects have not yet been directly demonstrated. Across such studies, the identity of the activating ligand is rarely resolved, which remains a limitation when attributing ZBP1-dependent injury specifically to Z-DNA.

## 10. Non-B DNA-Associated Genomic Instability in Metabolic Disease

**Figure 5:**
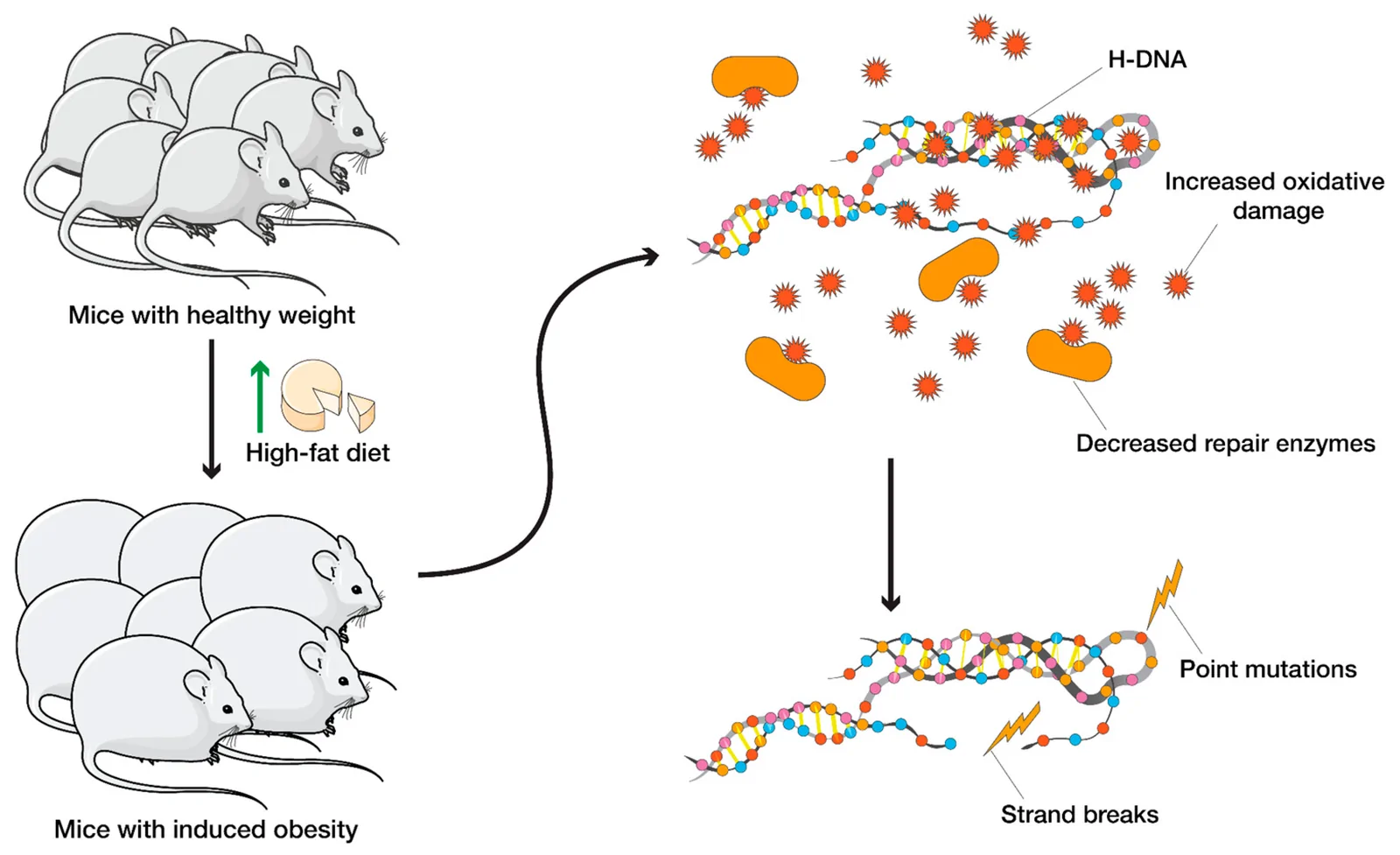
Diet-induced obesity in transgenic mice increases genomic instability of H-DNA-forming loci. Mice carrying chromosomally integrated mutation reporters containing either an H-DNA-forming sequence or a control B-DNA sequence were fed control or obesity-inducing high-fat diets. In obese mice, increased oxidative stress accompanied by decreased repair enzyme activity led to high genomic instability in the H-DNA-forming transgene, resulting in both point mutations and strand breaks.

Non-B DNA may contribute to metabolic disease by promoting DNA damage and mutations, while metabolic stress may further increase the instability of sequences favoring non-B DNA formation. H-DNA, an intramolecular triplex formed at mirror repeats [139], provides a direct example (Figure 5). In transgenic mice carrying either an H-DNA-forming sequence or a matched B-DNA control, high-fat-diet-induced obesity increased H-DNA-associated mutagenesis in the liver, brain, and testes [140] (Figure 5). This included both point mutations and large deletions, with the relative contribution of each varying between tissues. DNA breaks were also detected within and around the H-DNA-forming region in the obese mice, further supporting increased structural instability during metabolic stress [140] (Figure 5). H-DNA-forming sequences were also more susceptible to oxidative DNA damage, while DNA repair activity was reduced in tissues from obese mice [140,141]. These findings suggest that metabolic stress can increase damage and mutagenesis at non-B-DNA-forming sequences (Figure 5). In a related mouse reporter model, aging increased H-DNA-associated mutagenesis in the liver and spleen but reduced it in the brain [142]. This highlights age and tissue context as factors to consider when investigating how metabolic stress affects non-B DNA-associated instability.

Expanded repeats provide another connection to metabolic disease. In Friedreich’s ataxia, reduced *FXN* expression and frataxin deficiency can impair pancreatic β-cell function and contribute to diabetes [143,144]. Expanded GAA·TTC repeats within *FXN* can form triplex-associated structures that inhibit transcription, while R-loops also accumulate at these repeats [145,146]. These structures may therefore contribute to *FXN* silencing and its metabolic consequences, but their direct role in diabetes has not yet been established.

Non-B DNA formation has also been proposed to contribute to disease-associated mutations in *HNF1A* in monogenic diabetes MODY type 3 and *LDLR* in familial hypercholesterolemia through hairpin formation or strand slippage [147,148,149]. However, these mechanisms have not been experimentally demonstrated. Supporting the broader mutagenic potential of these sequences, aging increased instability at a short cruciform-forming inverted repeat in mice, including a marked increase in large deletions in the brain [150].

Overall, non-B DNA structures may contribute to metabolic disease through genomic instability and repeat-associated gene silencing. Emerging evidence further indicates that metabolic state can modulate genomic stability itself, with obesity increasing instability at an H-DNA-forming sequence and caloric restriction reducing somatic mutagenesis genome-wide [151]. Whether non-B DNA-forming loci are particularly sensitive to such metabolic modulation at endogenous genomic sites remains largely unexplored.

## 11. Therapeutic Perspectives: Targeting Non-B DNA in Metabolic Disease

Some of the structures described above have already been targeted with small molecules, providing early evidence that modulating non-B DNA can be metabolically beneficial. The i-motif-binding compound A22 stabilizes the *BCL2* promoter i-motif and, in fatty liver models, was shown to reduce hepatocyte apoptosis, inflammation, and fibrosis while improving glucose tolerance [72]. The G-quadruplex-stabilizing ligands pyridostatin and Braco-19 illustrate the opposite side of this coin, as structural stabilization is not uniformly beneficial: pyridostatin repressed *CES2* and increased hepatocyte triglyceride accumulation [67], while Braco-19 suppressed adipogenic differentiation and lipid accumulation [57]. These examples indicate that the same pharmacological strategy can be protective or detrimental depending on the target locus, underlining the need for selectivity.

More broadly, targeting non-B DNA has begun to move from concept toward the clinic, and most of this momentum has come from G-quadruplexes [152]. The G4-stabilizing ligand CX-5461 (pidnarulex) is selectively lethal in *BRCA1/2*-deficient, homologous-recombination-compromised tumors [153] and has completed a phase I trial in patients with DNA-repair-deficient solid cancers [154,155]. A second-generation G4 ligand, QN-302, has entered phase I testing in pancreatic cancer [156], and the medicinal-chemistry effort behind such molecules continues to expand [157]. Mechanistically, the cytotoxicity of G4 ligands has been linked to transcription-associated topoisomerase 2α activity and consequent DNA damage [158], highlighting that the same structures that act as endogenous mutation hotspots can also be utilized therapeutically.

Comparable, though earlier-stage, strategies are emerging for the other structures as well: i-motifs are increasingly considered as active regulatory elements rather than passive complementary structures [159], R-loop homeostasis can in principle be shifted pharmacologically, for example, by modulating the RNA helicases that resolve them [160], and the ZBP1/Z-nucleic acid sensing axis central to inflammatory cell death in metabolic tissues is itself being explored as a therapeutic target, with its clinical translation still considered challenging [161,162]. For repeat-associated structures, structure-directed oligonucleotides aimed at the triplex- and R-loop-forming GAA·TTC expansion can restore frataxin expression [163], offering a template of potential relevance to the β-cell dysfunction of Friedreich’s ataxia-associated diabetes.

A recurring obstacle, as the A22, pyridostatin, and Braco-19 examples make clear, is selectivity. Broadly stabilizing or disrupting a specific non-B DNA structure entails the risk of disturbing beneficial regulation. Locus-specific approaches, such as CRISPR-guided delivery of G4-binding proteins or ligands [164,165], may improve targeting precision. Ligands targeting higher-order rather than individual G-quadruplexes [166] may further support the selective interventions needed for metabolic applications.

## 12. Conclusions and Future Directions

Non-B DNA structures provide an additional layer of regulation connecting genome function to metabolic homeostasis. The studies discussed here link G4s and i-motifs to insulin- and lipid-related gene expression, R-loops to metabolic gene regulation and tissue injury, and mitochondrial non-B DNA to impaired respiration and inflammatory signaling. Repeat-associated structures provide further connections through genomic instability and gene silencing. These effects depend on the structure and its location: both excessive accumulation and loss of regulatory structures can contribute to disease. Conversely, metabolic stress can increase damage at non-B-DNA-forming sequences, suggesting that this relationship operates in both directions.

Fully elucidating these molecular relationships is challenging, due to the difficulty of detecting non-B DNA structures directly in vivo [6]. Computational tools and in vitro assays identify sequences capable of adopting non-B conformations but cannot account for the ionic, topological, and chromatin context of a living cell [167,168,169]. Most cell-based approaches rely on structure-specific antibodies, binding proteins, or catalytically inactive enzymes [170,171,172,173], which can exhibit binding biases, stabilize or unfold their targets [174,175,176], and cross-react with other nucleic acids, as reported for the RNA–DNA hybrid antibody S9.6 [177]. Because many protocols also require fixation or other preparation steps, the structures detected partly reflect the conditions of each assay [5]. In addition, most mapping approaches average signals across cell populations, obscuring the transient and heterogeneous formation of these structures at individual loci [178]. Live-cell sensors, structure-specific nanobodies and fluorescent probes, and in-cell NMR have begun to address these limitations [179,180,181,182], but they have so far been applied mainly in cultured cells.

Despite these difficulties, it remains a central priority to establish when and where these structures form in metabolic tissues. Direct measurements in pancreatic β cells, hepatocytes, adipocytes, and renal cells, together with corresponding gene expression and metabolic measurements at different ages, could help distinguish regulatory structures from those associated with cellular stress. Comparing disease etiology and progression would also help determine whether structural changes precede tissue dysfunction or arise as a consequence of injury. The evidence discussed here is weighted toward G4s, R-loops, and Z-DNA, reflecting both the current literature and the greater availability of structure-specific detection tools, whereas H-DNA, cruciforms, and slipped-strand structures remain comparatively unexplored in metabolic tissues.

Establishing causality will require interventions that distinguish the effects of a specific structure from the broader functions of its regulatory proteins or the compounds used to target it. This is particularly important for mitochondrial RNA-DNA hybrids and ZBP1-associated injury, where altered RNA processing or recognition of Z-RNA may contribute to the reported phenotypes. Testing whether formation of an endogenous structure restores metabolic function in vivo would strengthen the connection between structural mechanisms and disease.

As discussed above, the therapeutic targeting of non-B DNA is advancing rapidly, but its application to metabolic disease will require selectivity for the relevant structure, locus, and tissue, since broadly stabilizing or removing these structures may disrupt beneficial regulatory functions alongside detrimental ones. Defining which structural changes drive disease and which support adaptation or repair will be essential for evaluating non-B DNA as a therapeutic target in metabolic disorders. It would also be worthwhile to evaluate whether therapies already in wide use, including statins, incretin analogs, SGLT2 inhibitors, and bariatric surgery, alter non-B DNA landscapes, either as an unrecognized component of their benefit or as a consequence of the metabolic and transcriptional remodeling they induce.

## Data Availability

No new data were created or analyzed in this study. Data sharing is not applicable to this article.

## Abbreviations

The following abbreviations are used in this manuscript:

*ABCA1*	ATP-binding cassette subfamily A member 1
*ACBP*	Acyl-CoA-binding protein
*ACC1*	Acetyl-CoA carboxylase 1
ADAR1	Adenosine deaminase acting on RNA 1
AIRE	Autoimmune regulator
*AP2*	Adipocyte protein 2 (fatty acid-binding protein 4)
*BCL2*	B-cell lymphoma 2
*BRCA1/2*	Breast cancer gene 1/2
*BRG1*	Brahma-related gene 1
*CES2*	Carboxylesterase 2
cGAS	Cyclic GMP–AMP synthase
cGAS-STING	Cyclic GMP–AMP synthase–stimulator of interferon genes
D-loop	Displacement loop
*DSP*	Desmoplakin
*DSP-AS1*	Desmoplakin antisense RNA 1
EndoG	Endonuclease G
*FXN*	Frataxin
G4	G-quadruplex
GADD45α	Growth arrest and DNA damage-inducible protein 45 alpha
*GCK*	Glucokinase
HIF-1	Hypoxia-inducible factor 1
HLA	Human leukocyte antigen
*HNF1A*	Hepatocyte nuclear factor 1 homeobox A
HNF4α	Hepatocyte nuclear factor 4 alpha
ILPR	Insulin-linked polymorphic region
*INS*	Insulin
*Ins1*	Insulin 1 (mouse)
*Ins2*	Insulin 2 (mouse)
*LDLR*	Low-density lipoprotein receptor
*LEP*	Leptin
MAFLD	Metabolic dysfunction-associated fatty liver disease
MASLD	Metabolic dysfunction-associated steatotic liver disease
MASH	Metabolic dysfunction-associated steatohepatitis
*MICB*	MHC class I polypeptide-related sequence B
MODY	Maturity-onset diabetes of the young
mtDNA	Mitochondrial DNA
mtSSB	Mitochondrial single-stranded DNA-binding protein
NAD^+^	Nicotinamide adenine dinucleotide
NASH	Non-alcoholic steatohepatitis
NONO	Non-POU domain-containing octamer-binding protein
OriL	Origin of light-strand DNA replication
PDX1	Pancreatic and duodenal homeobox 1
*PLA2G6*	Phospholipase A2 group VI
*POLRMT*	Mitochondrial RNA polymerase
*PPARG*	Peroxisome proliferator-activated receptor gamma
RHIM	RIP homotypic interaction motif
RIPK1	Receptor-interacting serine/threonine-protein kinase 1
RIPK3	Receptor-interacting serine/threonine-protein kinase 3
*RNASEH1*	Ribonuclease H1
SGLT2	Sodium–glucose cotransporter 2
*SLC11A1*	Solute carrier family 11 member 1
SRSF1	Serine/arginine-rich splicing factor 1
SRSF3	Serine/arginine-rich splicing factor 3
SSB	Sjögren syndrome antigen B (La protein)
*STEAP4*	Six-transmembrane epithelial antigen of the prostate 4
SWI/SNF	Switch/sucrose non-fermentable
*TM6SF2*	Transmembrane 6 superfamily member 2
*UCP1*	Uncoupling protein 1
ZBP1	Z-DNA-binding protein 1

## References

[1] Watson J.D., Crick F.H. (1953). Molecular structure of nucleic acids; a structure for deoxyribose nucleic acid. Nature.

[2] Ghosh A., Bansal M. (2003). A glossary of DNA structures from A to Z. Acta Crystallogr. Sect. D Biol. Crystallogr..

[3] Kaushik M., Kaushik S., Roy K., Singh A., Mahendru S., Kumar M., Chaudhary S., Ahmed S., Kukreti S. (2016). A bouquet of DNA structures: Emerging diversity. Biochem. Biophys. Rep..

[4] Choi J., Majima T. (2011). Conformational changes of non-B DNA. Chem. Soc. Rev..

[5] Wang G., Vasquez K.M. (2023). Dynamic alternative DNA structures in biology and disease. Nat. Rev. Genet..

[6] Bochalis E., Dereki I., Wang G., Sgourou A., Vasquez K.M., Georgakopoulos-Soares I. (2026). Non-B DNA structures and their contributions to genetic diversity, aging, and disease. Nucleic Acids Res..

[7] Spiegel J., Adhikari S., Balasubramanian S. (2020). The Structure and Function of DNA G-Quadruplexes. Trends Chem..

[8] Day H.A., Pavlou P., Waller Z.A. (2014). I-Motif DNA: Structure, stability and targeting with ligands. Bioorg. Med. Chem..

[9] Krall J.B., Nichols P.J., Henen M.A., Vicens Q., Vögeli B. (2023). Structure and Formation of Z-DNA and Z-RNA. Molecules.

[10] Li X., Song G., Dou L., Yan S., Zhang M., Yuan W., Lai S., Jiang X., Li K., Sun K. (2021). The structure and unzipping behavior of dumbbell and hairpin DNA revealed by real-time nanopore sensing. Nanoscale.

[11] Mandal S., Selvam S., Cui Y., Hoque M.E., Mao H. (2018). Mechanical Cooperativity in DNA Cruciform Structures. Chemphyschem.

[12] Sinden R.R., Pytlos-Sinden M.J., Potaman V.N. (2007). Slipped strand DNA structures. Front. Biosci..

[13] McGinty R., Lyskova A., Mirkin S.M. (2025). The origin of mirror repeats in the human genome. Nucleic Acids Res..

[14] Belotserkovskii B.P., Tornaletti S., D’Souza A.D., Hanawalt P.C. (2018). R-loop generation during transcription: Formation, processing and cellular outcomes. DNA Repair.

[15] Svozil D., Kalina J., Omelka M., Schneider B. (2008). DNA conformations and their sequence preferences. Nucleic Acids Res..

[16] Pina A.F., Sousa S.F., Azevedo L., Carneiro J. (2022). Non-B DNA conformations analysis through molecular dynamics simulations. Biochim. Biophys. Acta Gen. Subj..

[17] Hong F., Schreck J.S., Šulc P. (2020). Understanding DNA interactions in crowded environments with a coarse-grained model. Nucleic Acids Res..

[18] Selvam S., Koirala D., Yu Z., Mao H. (2014). Quantification of topological coupling between DNA superhelicity and G-quadruplex formation. J. Am. Chem. Soc..

[19] Williams S.L., Casas-Delucchi C.S., Raguseo F., Guneri D., Li Y., Minamino M., Fletcher E.E., Yeeles J.T., Keyser U.F., Waller Z.A. (2023). Replication-induced DNA secondary structures drive fork uncoupling and breakage. EMBO J..

[20] Kouzine F., Wojtowicz D., Baranello L., Yamane A., Nelson S., Resch W., Kieffer-Kwon K.R., Benham C.J., Casellas R., Przytycka T.M. (2017). Permanganate/S1 Nuclease Footprinting Reveals Non-B DNA Structures with Regulatory Potential across a Mammalian Genome. Cell Syst..

[21] Georgakopoulos-Soares I., Chan C.S.Y., Ahituv N., Hemberg M. (2022). High-throughput techniques enable advances in the roles of DNA and RNA secondary structures in transcriptional and post-transcriptional gene regulation. Genome Biol..

[22] Mellor C., Perez C., Sale J.E. (2022). Creation and resolution of non-B-DNA structural impediments during replication. Crit. Rev. Biochem. Mol. Biol..

[23] Wulfridge P., Yan Q., Rell N., Doherty J., Jacobson S., Offley S., Deliard S., Feng K., Phillips-Cremins J.E., Gardini A. (2023). G-quadruplexes associated with R-loops promote CTCF binding. Mol. Cell.

[24] Huertas P., Aguilera A. (2003). Cotranscriptionally formed DNA:RNA hybrids mediate transcription elongation impairment and transcription-associated recombination. Mol. Cell.

[25] Georgakopoulos-Soares I., Parada G.E., Wong H.Y., Medhi R., Furlan G., Munita R., Miska E.A., Kwok C.K., Hemberg M. (2022). Alternative splicing modulation by G-quadruplexes. Nat. Commun..

[26] Georgakopoulos-Soares I., Morganella S., Jain N., Hemberg M., Nik-Zainal S. (2018). Noncanonical secondary structures arising from non-B DNA motifs are determinants of mutagenesis. Genome Res..

[27] Guiblet W.M., Cremona M.A., Harris R.S., Chen D., Eckert K.A., Chiaromonte F., Huang Y.F., Makova K.D. (2021). Non-B DNA: A major contributor to small- and large-scale variation in nucleotide substitution frequencies across the genome. Nucleic Acids Res..

[28] Wang G., Vasquez K.M. (2004). Naturally occurring H-DNA-forming sequences are mutagenic in mammalian cells. Proc. Natl. Acad. Sci. USA.

[29] Wang G., Christensen L.A., Vasquez K.M. (2006). Z-DNA-forming sequences generate large-scale deletions in mammalian cells. Proc. Natl. Acad. Sci. USA.

[30] Buisson R., Langenbucher A., Bowen D., Kwan E.E., Benes C.H., Zou L., Lawrence M.S. (2019). Passenger hotspot mutations in cancer driven by APOBEC3A and mesoscale genomic features. Science.

[31] Bacolla A., Tainer J.A., Vasquez K.M., Cooper D.N. (2016). Translocation and deletion breakpoints in cancer genomes are associated with potential non-B DNA-forming sequences. Nucleic Acids Res..

[32] Zewail-Foote M., Del Mundo I.M.A., Klattenhoff A.W., Vasquez K.M. (2025). Oxidative damage within alternative DNA structures results in aberrant mutagenic processing. Nucleic Acids Res..

[33] Simone R., Fratta P., Neidle S., Parkinson G.N., Isaacs A.M. (2015). G-quadruplexes: Emerging roles in neurodegenerative diseases and the non-coding transcriptome. FEBS Lett..

[34] Stein M., Eckert K.A. (2021). Impact of G-Quadruplexes and Chronic Inflammation on Genome Instability: Additive Effects during Carcinogenesis. Genes.

[35] D’Amico A.M., Li T.T., Vasquez K.M. (2024). Tissue-Specific Effects of Aging on Repeat-Mediated Mutation Hotspots In Vivo. Biomolecules.

[36] Guneri D., Alexandrou E., El Omari K., Dvořáková Z., Chikhale R.V., Pike D.T.S., Waudby C.A., Morris C.J., Haider S., Parkinson G.N. (2024). Structural insights into i-motif DNA structures in sequences from the insulin-linked polymorphic region. Nat. Commun..

[37] Nishino M., Ikegami H., Fujisawa T., Kawaguchi Y., Kawabata Y., Shintani M., Ono M., Ogihara T. (2005). Functional polymorphism in Z-DNA-forming motif of promoter of SLC11A1 gene and type 1 diabetes in Japanese subjects: Association study and meta-analysis. Metab. Clin. Exp..

[38] Wu P., Das M., Wang Y., Ji Y., Wu Y., Kumar D., Jih L.J., Webster N.J. (2026). SRSF3-TRIM28-MDC1 prevents DNA damage caused by R-loops in fatty liver disease in mice. JCI Insight.

[39] Rahman M.S., Hossain K.S., Das S., Kundu S., Adegoke E.O., Rahman M.A., Hannan M.A., Uddin M.J., Pang M.G. (2021). Role of Insulin in Health and Disease: An Update. Int. J. Mol. Sci..

[40] Carry P.M., Vanderlinden L.A., Johnson R.K., Dong F., Steck A.K., Frohnert B.I., Rewers M., Yang I.V., Kechris K., Norris J.M. (2020). DNA methylation near the INS gene is associated with INS genetic variation (rs689) and type 1 diabetes in the Diabetes Autoimmunity Study in the Young. Pediatr. Diabetes.

[41] Lew A., Rutter W.J., Kennedy G.C. (2000). Unusual DNA structure of the diabetes susceptibility locus IDDM2 and its effect on transcription by the insulin promoter factor Pur-1/MAZ. Proc. Natl. Acad. Sci. USA.

[42] Dhakal S., Schonhoft J.D., Koirala D., Yu Z., Basu S., Mao H. (2010). Coexistence of an ILPR i-motif and a partially folded structure with comparable mechanical stability revealed at the single-molecule level. J. Am. Chem. Soc..

[43] Dhakal S., Yu Z., Konik R., Cui Y., Koirala D., Mao H. (2012). G-quadruplex and i-motif are mutually exclusive in ILPR double-stranded DNA. Biophys. J..

[44] Catasti P., Chen X., Moyzis R.K., Bradbury E.M., Gupta G. (1996). Structure-function correlations of the insulin-linked polymorphic region. J. Mol. Biol..

[45] Schonhoft J.D., Das A., Achamyeleh F., Samdani S., Sewell A., Mao H., Basu S. (2010). ILPR repeats adopt diverse G-quadruplex conformations that determine insulin binding. Biopolymers.

[46] Paritala H., Firestine S.M. (2010). Characterization of insulin ILPR sequences for their ability to adopt a G-quadruplex structure. Nucleosides Nucleotides Nucleic Acids.

[47] Guneri D., Morris C.J., Ding Y., Craggs T.D., Smith S.S., Waller Z.A.E. (2026). I-Motif, not G-quadruplex, stability regulates insulin expression. Nucleic Acids Res..

[48] Connor A.C., Frederick K.A., Morgan E.J., McGown L.B. (2006). Insulin capture by an insulin-linked polymorphic region G-quadruplex DNA oligonucleotide. J. Am. Chem. Soc..

[49] Xiao J., McGown L.B. (2009). Mass spectrometric determination of ILPR G-quadruplex binding sites in insulin and IGF-2. J. Am. Soc. Mass Spectrom..

[50] Wickhorst P.J., Ihmels H., Paululat T. (2021). Studies on the Interactions of 3,11-Difluoro-6,8,13-trimethyl-8*H*-quino [4,3,2-*kl*]acridinium and Insulin with the Quadruplex-Forming Oligonucleotide Sequence a2 from the Insulin-Linked Polymorphic Region. Molecules.

[51] Lages A., Proud C.G., Holloway J.W., Vorechovsky I. (2019). Thioflavin T Monitoring of Guanine Quadruplex Formation in the rs689-Dependent INS Intron 1. Mol. Ther. Nucleic Acids.

[52] Královicová J., Gaunt T.R., Rodriguez S., Wood P.J., Day I.N., Vorechovsky I. (2006). Variants in the human insulin gene that affect pre-mRNA splicing: Is -23HphI a functional single nucleotide polymorphism at IDDM2?. Diabetes.

[53] Lahnsteiner A., Ellmer V., Oberlercher A., Liutkeviciute Z., Schönauer E., Paulweber B., Aigner E., Risch A. (2024). G-quadruplex forming regions in GCK and TM6SF2 are targets for differential DNA methylation in metabolic disease and hepatocellular carcinoma patients. Sci. Rep..

[54] Xiang L., Niu K., Peng Y., Zhang X., Li X., Ye R., Yu G., Ye G., Xiang H., Song Q. (2022). DNA G-quadruplex structure participates in regulation of lipid metabolism through acyl-CoA binding protein. Nucleic Acids Res..

[55] Xiao C.D., Jia M.H., Zhong M.Q., Xu Y., Yu Z.T., He Z.Y., Lu X., Zhang Y., Zhou X., Fu L.Y. (2023). Unveiling the role of G-quadruplex structure in promoter region: Regulation of ABCA1 expression in macrophages possibly via NONO protein recruitment. Int. J. Biol. Macromol..

[56] Zhao Y., Uhler J.P. (2018). Identification of a G-quadruplex forming sequence in the promoter of UCP1. Acta Biochim. Biophys. Sin..

[57] Ambrosio M.R., Migliaccio T., Napolitano F., Di Somma S., Maneli G., Amato J., Pagano B., Randazzo A., Portella G., Formisano P. (2023). Targeting G-quadruplex motifs interferes with differentiation of adipose-derived mesenchymal stem cells. Stem Cell Res. Ther..

[58] Abu Aqel Y., Alnesf A., Aigha I.I., Islam Z., Kolatkar P.R., Teo A., Abdelalim E.M. (2024). Glucokinase (GCK) in diabetes: From molecular mechanisms to disease pathogenesis. Cell. Mol. Biol. Lett..

[59] Zhang X., Lau H.C., Ha S., Liu C., Liang C., Lee H.W., Ng Q.W., Zhao Y., Ji F., Zhou Y. (2025). Intestinal TM6SF2 protects against metabolic dysfunction-associated steatohepatitis through the gut-liver axis. Nat. Metab..

[60] Al-Nakeb M., Haep N. (2026). TM6SF2 in Hepatic Lipid Metabolism and Chronic Liver Disease. Livers.

[61] Duman C., Yaqubi K., Hoffmann A., Acikgöz A.A., Korshunov A., Bendszus M., Herold-Mende C., Liu H.K., Alfonso J. (2019). Acyl-CoA-Binding Protein Drives Glioblastoma Tumorigenesis by Sustaining Fatty Acid Oxidation. Cell Metab..

[62] Babashamsi M.M., Koukhaloo S.Z., Halalkhor S., Salimi A., Babashamsi M. (2019). ABCA1 and metabolic syndrome; a review of the ABCA1 role in HDL-VLDL production, insulin-glucose homeostasis, inflammation and obesity. Diabetes Metab. Syndr..

[63] Fedorenko A., Lishko P.V., Kirichok Y. (2012). Mechanism of fatty-acid-dependent UCP1 uncoupling in brown fat mitochondria. Cell.

[64] Tong L., Harwood H.J. (2006). Acetyl-coenzyme A carboxylases: Versatile targets for drug discovery. J. Cell. Biochem..

[65] Kaulage M., Maji B., Bhat J., Iwasaki Y., Chatterjee S., Bhattacharya S., Muniyappa K. (2016). Discovery and Structural Characterization of G-quadruplex DNA in Human Acetyl-CoA Carboxylase Gene Promoters: Its Role in Transcriptional Regulation and as a Therapeutic Target for Human Disease. J. Med. Chem..

[66] Chalhoub G., Kolleritsch S., Maresch L.K., Taschler U., Pajed L., Tilp A., Eisner H., Rosina P., Kien B., Radner F.P.W. (2021). Carboxylesterase 2 proteins are efficient diglyceride and monoglyceride lipases possibly implicated in metabolic disease. J. Lipid Res..

[67] Kuroda S., Nakashima S., Takemoto S., Nakano M., Higuchi Y., Uehara S., Yoneda N., Suemizu H., Nakajima M., Fukami T. (2026). Regulatory impact of G-quadruplexes in the human carboxylesterase 2 gene on drug and lipid metabolism. Drug Metab. Pharmacokinet..

[68] Fan J., Zhang X., Zhang J., Zhao T., Burley S.K., Zheng X.F.S. (2026). PDX1 phosphorylation at S61 by mTORC1 links nutrient signaling to β cell function and metabolic disease. Cell Rep..

[69] Ebrahim N., Shakirova K., Dashinimaev E. (2022). PDX1 is the cornerstone of pancreatic β-cell functions and identity. Front. Mol. Biosci..

[70] Lahnsteiner A., Ellmer V., Schönauer E., Huber J., Lexa M., Wiederstein M., Risch A. (2026). Metabolic disease variants rewire gene regulation through disruption of DNA G-quadruplex structures. Genome Biol..

[71] Wang Z., Tang M., Konopleva M. (2026). Anti-apoptotic BCL-2 family proteins: From regulatory networks to therapeutic targeting. Oncogenesis.

[72] Li X., Wang J., Gong X., Zhang M., Kang S., Shu B., Wei Z., Huang Z.S., Li D. (2020). Upregulation of BCL-2 by acridone derivative through gene promoter i-motif for alleviating liver damage of NAFLD/NASH. Nucleic Acids Res..

[73] Kaulage M.H., Bhattacharya S., Muniyappa K. (2018). Structural Characterization of i-Motif Structure in the Human Acetyl-CoA Carboxylase 1 Gene Promoters and Their Role in the Regulation of Gene Expression. ChemBioChem.

[74] Yang S., Winstone L., Mondal S., Wu Y. (2023). Helicases in R-loop Formation and Resolution. J. Biol. Chem..

[75] Kim A., Wang G.G. (2021). R-loop and its functions at the regulatory interfaces between transcription and (epi)genome. Biochim. Biophys. Acta Gene Regul. Mech..

[76] Brickner J.R., Garzon J.L., Cimprich K.A. (2022). Walking a tightrope: The complex balancing act of R-loops in genome stability. Mol. Cell.

[77] Pezone A., Olivieri F., Napoli M.V., Procopio A., Avvedimento E.V., Gabrielli A. (2023). Inflammation and DNA damage: Cause, effect or both. Nat. Rev. Rheumatol..

[78] Crossley M.P., Song C., Bocek M.J., Choi J.H., Kousouros J.N., Sathirachinda A., Lin C., Brickner J.R., Bai G., Lans H. (2023). R-loop-derived cytoplasmic RNA-DNA hybrids activate an immune response. Nature.

[79] Weinreb J.T., Ghazale N., Pradhan K., Gupta V., Potts K.S., Tricomi B., Daniels N.J., Padgett R.A., De Oliveira S., Verma A. (2021). Excessive R-loops trigger an inflammatory cascade leading to increased HSPC production. Dev. Cell.

[80] Zannini L., Cardano M., Liberi G., Buscemi G. (2024). R-loops and impaired autophagy trigger cGAS-dependent inflammation via micronuclei formation in Senataxin-deficient cells. Cell. Mol. Life Sci. CMLS.

[81] Li X.Q., Zhang J.X., Li L., Wu Q.Y., Ruan X.Z., Chen P.P., Ma K.L. (2025). Deficiency of Growth Arrest and DNA Damage-Inducible 45 α -R-Loop Pathway and Kidney Injury in Diabetic Nephropathy. J. Am. Soc. Nephrol. JASN.

[82] Rayego-Mateos S., Rodrigues-Diez R.R., Fernandez-Fernandez B., Mora-Fernández C., Marchant V., Donate-Correa J., Navarro-González J.F., Ortiz A., Ruiz-Ortega M. (2023). Targeting inflammation to treat diabetic kidney disease: The road to 2030. Kidney Int..

[83] Martinez Leon V., Hilburg R., Susztak K. (2026). Mechanisms of diabetic kidney disease and established and emerging treatments. Nat. Rev. Endocrinol..

[84] Xing J., Wang S. (2026). SSB deficiency-induced R-loop accumulation triggers podocyte inflammation in DKD. Front. Immunol..

[85] Kazmi I., Al-Abbasi F.A., Zeyadi M., Rafeeq M., Habib A.H., Iqbal J., Alzarea S.I., Alsaidan O.A., Nadeem M.S. (2025). Translational potential of GADD45α: Biomarker and therapeutic target in age-associated neurodegeneration and longevity. Biogerontology.

[86] Tamura T., Chiba J. (2009). STEAP4 regulates focal adhesion kinase activation and CpG motifs within STEAP4 promoter region are frequently methylated in DU145, human androgen-independent prostate cancer cells. Int. J. Mol. Med..

[87] Ten Freyhaus H., Calay E.S., Yalcin A., Vallerie S.N., Yang L., Calay Z.Z., Saatcioglu F., Hotamisligil G.S. (2012). Stamp2 controls macrophage inflammation through nicotinamide adenine dinucleotide phosphate homeostasis and protects against atherosclerosis. Cell Metab..

[88] Han D.D., Jew G., Kaproth-Joslin K. (2026). Desmoplakin Cardiomyopathy. Int. J. Cardiovasc. Imaging.

[89] Yang C., Lian H., Luo H., Song C., Lin J., Liang Z., Yang Y., Hong X., Li S., Chen Y. (2025). The Diminution of R-Loops Generated by LncRNA DSP-AS1 Inhibits DSP Gene Transcription to Impede the Re-Epithelialization During Diabetic Wound Healing. Adv. Sci..

[90] Arif W., Mathur B., Saikali M.F., Chembazhi U.V., Toohill K., Song Y.J., Hao Q., Karimi S., Blue S.M., Yee B.A. (2023). Splicing factor SRSF1 deficiency in the liver triggers NASH-like pathology and cell death. Nat. Commun..

[91] Kumar D., Das M., Sauceda C., Ellies L.G., Kuo K., Parwal P., Kaur M., Jih L., Bandyopadhyay G.K., Burton D. (2019). Degradation of splicing factor SRSF3 contributes to progressive liver disease. J. Clin. Investig..

[92] Fu J., Wei Y., Yang Y., Yang X., Ouyang T., Wang X., Chen S., Liu Z., Su Y., Fu J. (2025). Intranuclear paraspeckle-circular RNA TACC3 assembly forms RNA-DNA hybrids to facilitate MASH-related hepatocellular carcinoma growth in an m6A-dependent manner. Cancer Commun..

[93] Baek H., Park S.U., Kim J. (2023). Emerging role for R-loop formation in hepatocellular carcinoma. Genes Genom..

[94] Yang W.Y., Saaoud F., Xu K., Ben Issa M., Lu Y., Shao Y., Han B., Wang X., Jiang X., Wu S. (2026). Reduced R-loop abundance at proinflammatory loci: A shared epigenetic mechanism in inflammatory and metabolic diseases. Front. Cardiovasc. Med..

[95] Debnath T.K., Abell N.S., Li Y.R., Devanathan S.K., Navedo E., Xhemalçe B. (2025). NAT10 and N4-acetylcytidine restrain R-loop levels and related inflammatory responses. Sci. Adv..

[96] Yang X., Li G., Lou P., Zhang M., Yao K., Xiao J., Chen Y., Xu J., Tian S., Deng M. (2024). Excessive nucleic acid R-loops induce mitochondria-dependent epithelial cell necroptosis and drive spontaneous intestinal inflammation. Proc. Natl. Acad. Sci. USA.

[97] Damas J., Carneiro J., Gonçalves J., Stewart J.B., Samuels D.C., Amorim A., Pereira F. (2012). Mitochondrial DNA deletions are associated with non-B DNA conformations. Nucleic Acids Res..

[98] Fusté J.M., Wanrooij S., Jemt E., Granycome C.E., Cluett T.J., Shi Y., Atanassova N., Holt I.J., Gustafsson C.M., Falkenberg M. (2010). Mitochondrial RNA polymerase is needed for activation of the origin of light-strand DNA replication. Mol. Cell.

[99] Wanrooij S., Miralles Fusté J., Stewart J.B., Wanrooij P.H., Samuelsson T., Larsson N.G., Gustafsson C.M., Falkenberg M. (2012). In vivo mutagenesis reveals that OriL is essential for mitochondrial DNA replication. EMBO Rep..

[100] Miralles Fusté J., Shi Y., Wanrooij S., Zhu X., Jemt E., Persson Ö., Sabouri N., Gustafsson C.M., Falkenberg M. (2014). In vivo occupancy of mitochondrial single-stranded DNA binding protein supports the strand displacement mode of DNA replication. PLoS Genet..

[101] Wang W., Li J., Tan J., Wang M., Yang J., Zhang Z.M., Li C., Basnakian A.G., Tang H.W., Perrimon N. (2021). Endonuclease G promotes autophagy by suppressing mTOR signaling and activating the DNA damage response. Nat. Commun..

[102] Xu X., Penjweini R., Székvölgyi L., Karányi Z., Heckel A.M., Gurusamy D., Varga D., Yang S., Brown A.L., Cui W. (2025). Endonuclease G promotes hepatic mitochondrial respiration by selectively increasing mitochondrial tRNAThr production. Proc. Natl. Acad. Sci. USA.

[103] Reyes A., Melchionda L., Nasca A., Carrara F., Lamantea E., Zanolini A., Lamperti C., Fang M., Zhang J., Ronchi D. (2015). RNASEH1 Mutations Impair mtDNA Replication and Cause Adult-Onset Mitochondrial Encephalomyopathy. Am. J. Hum. Genet..

[104] Bharti S.K., Sommers J.A., Zhou J., Kaplan D.L., Spelbrink J.N., Mergny J.L., Brosh R.M. (2014). DNA sequences proximal to human mitochondrial DNA deletion breakpoints prevalent in human disease form G-quadruplexes, a class of DNA structures inefficiently unwound by the mitochondrial replicative Twinkle helicase. J. Biol. Chem..

[105] Dong D.W., Pereira F., Barrett S.P., Kolesar J.E., Cao K., Damas J., Yatsunyk L.A., Johnson F.B., Kaufman B.A. (2014). Association of G-quadruplex forming sequences with human mtDNA deletion breakpoints. BMC Genom..

[106] Butler T.J., Estep K.N., Sommers J.A., Maul R.W., Moore A.Z., Bandinelli S., Cucca F., Tuke M.A., Wood A.R., Bharti S.K. (2020). Mitochondrial genetic variation is enriched in G-quadruplex regions that stall DNA synthesis in vitro. Hum. Mol. Genet..

[107] Doimo M., Chaudhari N., Abrahamsson S., L’Hôte V., Nguyen T.V.H., Berner A., Ndi M., Abrahamsson A., Das R.N., Aasumets K. (2023). Enhanced mitochondrial G-quadruplex formation impedes replication fork progression leading to mtDNA loss in human cells. Nucleic Acids Res..

[108] Falabella M., Kolesar J.E., Wallace C., de Jesus D., Sun L., Taguchi Y.V., Wang C., Wang T., Xiang I.M., Alder J.K. (2019). G-quadruplex dynamics contribute to regulation of mitochondrial gene expression. Sci. Rep..

[109] Naeem M.M., Maheshan R., Costford S.R., Wahedi A., Trajkovski M., Plavec J., Yatsunyk L.A., Ciesielski G.L., Kaufman B.A., Sondheimer N. (2019). G-quadruplex-mediated reduction of a pathogenic mitochondrial heteroplasmy. Hum. Mol. Genet..

[110] Liu Y., Hu C., Luo S., Han Y., Li L., Wei L., Chen W., Yang M. (2026). S14G-humanin (HNG) ameliorates diabetic nephropathy tubular injury by inhibiting Z-DNA/ZBP1-mediated necroptosis. Eur. J. Pharmacol..

[111] Szczesny B., Marcatti M., Ahmad A., Montalbano M., Brunyánszki A., Bibli S.I., Papapetropoulos A., Szabo C. (2018). Mitochondrial DNA damage and subsequent activation of Z-DNA binding protein 1 links oxidative stress to inflammation in epithelial cells. Sci. Rep..

[112] Chen C., Xie J., Chen Z., Ye K., Wu C., Dai X., Yuan Y., Lin Y., Wang Y., Chen H. (2025). Role of Z-DNA Binding Protein 1 Sensing Mitochondrial Z-DNA and Triggering Necroptosis in Oxalate-Induced Acute Kidney Injury. J. Am. Soc. Nephrol. JASN.

[113] Hu Z., Ma Y., Wang Y., Wang J., Liu X., Chen C., Zhu W., Qi X., Wu Y. (2026). ZBP1 Mediates Renal Tubular Injury in Diabetic Nephropathy Through RIPK3-mediated Necroptosis. Inflammation.

[114] Liu R., Cao H., Zhang S., Cai M., Zou T., Wang G., Zhang D., Wang X., Xu J., Deng S. (2024). ZBP1-mediated apoptosis and inflammation exacerbate steatotic liver ischemia/reperfusion injury. J. Clin. Investig..

[115] Zhang T., Yin C., Fedorov A., Qiao L., Bao H., Beknazarov N., Wang S., Gautam A., Williams R.M., Crawford J.C. (2022). ADAR1 masks the cancer immunotherapeutic promise of ZBP1-driven necroptosis. Nature.

[116] Duan J., Cao Z., Zhou Z., Huang X., Zhao J., Huang Y., Huang T., Meng S., Chen X., Hong T. (2026). Mitochondrial dysfunction drives ZBP1-mediated PANoptosis to increase the susceptibility of heart failure with preserved ejection fraction-associated atrial fibrillation. J. Adv. Res..

[117] Nairz M., Fritsche G., Crouch M.L., Barton H.C., Fang F.C., Weiss G. (2009). SLC11A1 limits intracellular growth of Salmonella enterica sv. Typhimurium by promoting macrophage immune effector functions and impairing bacterial iron acquisition. Cell. Microbiol..

[118] Kissler S., Stern P., Takahashi K., Hunter K., Peterson L.B., Wicker L.S. (2006). In vivo RNA interference demonstrates a role for Nramp1 in modifying susceptibility to type 1 diabetes. Nat. Genet..

[119] Xu Y.Z., Thuraisingam T., Marino R., Radzioch D. (2011). Recruitment of SWI/SNF complex is required for transcriptional activation of the SLC11A1 gene during macrophage differentiation of HL-60 cells. J. Biol. Chem..

[120] Searle S., Blackwell J.M. (1999). Evidence for a functional repeat polymorphism in the promoter of the human NRAMP1 gene that correlates with autoimmune versus infectious disease susceptibility. J. Med. Genet..

[121] Bayele H.K., Peyssonnaux C., Giatromanolaki A., Arrais-Silva W.W., Mohamed H.S., Collins H., Giorgio S., Koukourakis M., Johnson R.S., Blackwell J.M. (2007). HIF-1 regulates heritable variation and allele expression phenotypes of the macrophage immune response gene SLC11A1 from a Z-DNA forming microsatellite. Blood.

[122] Yang J.H., Downes K., Howson J.M., Nutland S., Stevens H.E., Walker N.M., Todd J.A. (2011). Evidence of association with type 1 diabetes in the SLC11A1 gene region. BMC Med. Genet..

[123] Fang Y., Bansal K., Mostafavi S., Benoist C., Mathis D. (2024). AIRE relies on Z-DNA to flag gene targets for thymic T cell tolerization. Nature.

[124] Fan Y., Rudert W.A., Grupillo M., He J., Sisino G., Trucco M. (2009). Thymus-specific deletion of insulin induces autoimmune diabetes. EMBO J..

[125] DeAntoneo C., Herbert A., Balachandran S. (2023). Z-form nucleic acid-binding protein 1 (ZBP1) as a sensor of viral and cellular Z-RNAs: Walking the razor’s edge. Curr. Opin. Immunol..

[126] Thapa R.J., Ingram J.P., Ragan K.B., Nogusa S., Boyd D.F., Benitez A.A., Sridharan H., Kosoff R., Shubina M., Landsteiner V.J. (2016). DAI Senses Influenza A Virus Genomic RNA and Activates RIPK3-Dependent Cell Death. Cell Host Microbe.

[127] Zhang T., Yin C., Boyd D.F., Quarato G., Ingram J.P., Shubina M., Ragan K.B., Ishizuka T., Crawford J.C., Tummers B. (2020). Influenza Virus Z-RNAs Induce ZBP1-Mediated Necroptosis. Cell.

[128] Jiao H., Wachsmuth L., Wolf S., Lohmann J., Nagata M., Kaya G.G., Oikonomou N., Kondylis V., Rogg M., Diebold M. (2022). ADAR1 averts fatal type I interferon induction by ZBP1. Nature.

[129] Hubbard N.W., Ames J.M., Maurano M., Chu L.H., Somfleth K.Y., Gokhale N.S., Werner M., Snyder J.M., Lichauco K., Savan R. (2022). ADAR1 mutation causes ZBP1-dependent immunopathology. Nature.

[130] de Reuver R., Verdonck S., Dierick E., Nemegeer J., Hessmann E., Ahmad S., Jans M., Blancke G., Van Nieuwerburgh F., Botzki A. (2022). ADAR1 prevents autoinflammation by suppressing spontaneous ZBP1 activation. Nature.

[131] Rice G.I., Kasher P.R., Forte G.M., Mannion N.M., Greenwood S.M., Szynkiewicz M., Dickerson J.E., Bhaskar S.S., Zampini M., Briggs T.A. (2012). Mutations in ADAR1 cause Aicardi-Goutières syndrome associated with a type I interferon signature. Nat. Genet..

[132] Jiao H., Wachsmuth L., Kumari S., Schwarzer R., Lin J., Eren R.O., Fisher A., Lane R., Young G.R., Kassiotis G. (2020). Z-nucleic-acid sensing triggers ZBP1-dependent necroptosis and inflammation. Nature.

[133] Devos M., Tanghe G., Gilbert B., Dierick E., Verheirstraeten M., Nemegeer J., de Reuver R., Lefebvre S., De Munck J., Rehwinkel J. (2020). Sensing of endogenous nucleic acids by ZBP1 induces keratinocyte necroptosis and skin inflammation. J. Exp. Med..

[134] Lei Y., VanPortfliet J.J., Chen Y.F., Bryant J.D., Li Y., Fails D., Torres-Odio S., Ragan K.B., Deng J., Mohan A. (2023). Cooperative sensing of mitochondrial DNA by ZBP1 and cGAS promotes cardiotoxicity. Cell.

[135] Hotamisligil G.S. (2017). Inflammation, metaflammation and immunometabolic disorders. Nature.

[136] Run Y., Tavakoli M., Zhang Y., Vasquez K.M., Zhang W. (2026). Formation and biological implications of Z-DNA. Trends Genet..

[137] Song Q., Fan Y., Zhang H., Wang N. (2024). Z-DNA binding protein 1 orchestrates innate immunity and inflammatory cell death. Cytokine Growth Factor Rev..

[138] Chiang C., Li Y., Ng S.K. (2021). The Role of the Z-DNA Binding Domain in Innate Immunity and Stress Granules. Front. Immunol..

[139] Jain A., Wang G., Vasquez K.M. (2008). DNA triple helices: Biological consequences and therapeutic potential. Biochimie.

[140] Kompella P., Wang G., Durrett R.E., Lai Y., Marin C., Liu Y., Habib S.L., DiGiovanni J., Vasquez K.M. (2024). Obesity increases genomic instability at DNA repeat-mediated endogenous mutation hotspots. Nat. Commun..

[141] Zewail-Foote M., Klattenhoff A.W., Vasquez K.M. (2026). DNA Structure-Dependent Enrichment of Oxidative Lesions. Chem. Res. Toxicol..

[142] D’Amico A.M., Li T.T., Wylie D., Wang G., Vasquez K.M. (2025). Aging alters genomic instability at endogenous mutation hotspots in mice. Sci. Rep..

[143] Ristow M., Mulder H., Pomplun D., Schulz T.J., Müller-Schmehl K., Krause A., Fex M., Puccio H., Müller J., Isken F. (2003). Frataxin deficiency in pancreatic islets causes diabetes due to loss of beta cell mass. J. Clin. Investig..

[144] Cnop M., Igoillo-Esteve M., Rai M., Begu A., Serroukh Y., Depondt C., Musuaya A.E., Marhfour I., Ladrière L., Moles Lopez X. (2012). Central role and mechanisms of β-cell dysfunction and death in friedreich ataxia-associated diabetes. Ann. Neurol..

[145] Sakamoto N., Chastain P.D., Parniewski P., Ohshima K., Pandolfo M., Griffith J.D., Wells R.D. (1999). Sticky DNA: Self-association properties of long GAA.TTC repeats in R.R.Y triplex structures from Friedreich’s ataxia. Mol. Cell.

[146] Groh M., Lufino M.M., Wade-Martins R., Gromak N. (2014). R-loops associated with triplet repeat expansions promote gene silencing in Friedreich ataxia and fragile X syndrome. PLoS Genet..

[147] Ellard S., Bulman M.P., Frayling T.M., Shepherd M., Hattersley A.T. (2000). Proposed mechanism for a novel insertion/deletion frameshift mutation (I414G415ATCG-->CCA) in the hepatocyte nuclear factor 1 alpha (HNF-1 alpha) gene which causes maturity-onset diabetes of the young (MODY). Hum. Mutat..

[148] Chae J.J., Kim S.H., Kim U.K., Han K.H., Kim H.S., Kastner D.L., Namkoong Y., Park Y.B., Lee C.C. (1999). Three novel small deletion mutations of the LDL receptor gene in Korean patients with familial hypercholesterolemia. Clin. Genet..

[149] Yamakawa-Kobayashi K., Kobayashi T., Yanagi H., Shimakura Y., Satoh J., Hamaguchi H. (1994). A novel complex mutation in the LDL receptor gene probably caused by the simultaneous occurrence of deletion and insertion in the same region. Hum. Genet..

[150] Mandke P., Kompella P., Wang G., Vasquez K.M. (2025). Aging Increases Short, Inverted Repeat-Mediated Genomic Instability In Vivo. Aging Cell.

[151] Grońska-Pęski M., Acosta-Rodríguez V., Srinivasa A., Garrido A., Picciotto S., Green C.B., Takahashi J.S., Shoag J.E., Evrony G.D. (2026). Caloric restriction modulates genome-wide somatic mutation in mice. Cell.

[152] Balasubramanian S., Hurley L.H., Neidle S. (2011). Targeting G-quadruplexes in gene promoters: A novel anticancer strategy?. Nat. Rev. Drug Discov..

[153] Xu H., Di Antonio M., McKinney S., Mathew V., Ho B., O’Neil N.J., Santos N.D., Silvester J., Wei V., Garcia J. (2017). CX-5461 is a DNA G-quadruplex stabilizer with selective lethality in BRCA1/2 deficient tumours. Nat. Commun..

[154] Hilton J., Gelmon K., Bedard P.L., Tu D., Xu H., Tinker A.V., Goodwin R., Laurie S.A., Jonker D., Hansen A.R. (2022). Results of the phase I CCTG IND.231 trial of CX-5461 in patients with advanced solid tumors enriched for DNA-repair deficiencies. Nat. Commun..

[155] Li H.X., He Y.M., Fei J., Guo M., Zeng C., Yan P.J., Xu Y., Qin G., Teng F.Y. (2025). The G-quadruplex ligand CX-5461: An innovative candidate for disease treatment. J. Transl. Med..

[156] Neidle S. (2024). A Phenotypic Approach to the Discovery of Potent G-Quadruplex Targeted Drugs. Molecules.

[157] Mendes E., Aljnadi I.M., Bahls B., Victor B.L., Paulo A. (2022). Major Achievements in the Design of Quadruplex-Interactive Small Molecules. Pharmaceuticals.

[158] Bossaert M., Pipier A., Riou J.F., Noirot C., Nguyên L.T., Serre R.F., Bouchez O., Defrancq E., Calsou P., Britton S. (2021). Transcription-associated topoisomerase 2α (TOP2A) activity is a major effector of cytotoxicity induced by G-quadruplex ligands. eLife.

[159] Brown S.L., Kendrick S. (2021). The i-Motif as a Molecular Target: More Than a Complementary DNA Secondary Structure. Pharmaceuticals.

[160] Herrera K., Takasaki K., Murayama T. (2026). Targeting R-loops: Diverse RNA helicases in R-loop resolution and their potential as targets for cancer therapy. Front. Cell Dev. Biol..

[161] Huang Y., Wang L., Zhu Y., Li X., Dai Y., He G., Jiang X. (2025). Z-DNA-binding protein 1-mediated programmed cell death: Mechanisms and therapeutic implications. Chin. Med. J..

[162] Triantafillidis J.K., Karakatsanis S. (2026). Treatment of Inflammatory Bowel Disease with Drugs Targeting PANoptosis: A Comprehensive Review. Biomedicines.

[163] Mozafari N., Milagres S., Umek T., Rocha C.S.J., Vargiu C.M., Freyberger F., Saher O., Napierala M., Napierala J.S., Blomberg P. (2025). Anti-gene oligonucleotides targeting Friedreich’s ataxia expanded GAA⋅TTC repeats increase Frataxin expression. Mol. Ther. Nucleic Acids.

[164] Nuccio S.P., Cadoni E., Nikoloudaki R., Galli S., Ler A.J., Sanchez-Cabanillas C., Maher T.E., Fan E., Guneri D., Flint G. (2025). Chemically modified CRISPR-Cas9 enables targeting of individual G-quadruplex and i-motif structures, revealing ligand-dependent transcriptional perturbation. Nat. Commun..

[165] Qin G., Liu Z., Yang J., Liao X., Zhao C., Ren J., Qu X. (2024). Targeting specific DNA G-quadruplexes with CRISPR-guided G-quadruplex-binding proteins and ligands. Nat. Cell Biol..

[166] Monsen R.C. (2023). Higher-order G-quadruplexes in promoters are untapped drug targets. Front. Chem..

[167] Brázda V., Kolomazník J., Lýsek J., Bartas M., Fojta M., Šťastný J., Mergny J.L. (2019). G4Hunter web application: A web server for G-quadruplex prediction. Bioinformatics.

[168] Wang G., Mouratidis I., Provatas K., Chantzi N., Patsakis M., Georgakopoulos-Soares I., Vasquez K.M. (2025). ZSeeker: An optimized algorithm for Z-DNA detection in genomic sequences. Brief. Bioinform..

[169] Jenjaroenpun P., Wongsurawat T., Yenamandra S.P., Kuznetsov V.A. (2015). QmRLFS-finder: A model, web server and stand-alone tool for prediction and analysis of R-loop forming sequences. Nucleic Acids Res..

[170] Biffi G., Tannahill D., McCafferty J., Balasubramanian S. (2013). Quantitative visualization of DNA G-quadruplex structures in human cells. Nat. Chem..

[171] Zeraati M., Langley D.B., Schofield P., Moye A.L., Rouet R., Hughes W.E., Bryan T.M., Dinger M.E., Christ D. (2018). I-motif DNA structures are formed in the nuclei of human cells. Nat. Chem..

[172] Ha S.C., Kim D., Hwang H.Y., Rich A., Kim Y.G., Kim K.K. (2008). The crystal structure of the second Z-DNA binding domain of human DAI (ZBP1) in complex with Z-DNA reveals an unusual binding mode to Z-DNA. Proc. Natl. Acad. Sci. USA.

[173] Chen L., Chen J.Y., Zhang X., Gu Y., Xiao R., Shao C., Tang P., Qian H., Luo D., Li H. (2017). R-ChIP Using Inactive RNase H Reveals Dynamic Coupling of R-loops with Transcriptional Pausing at Gene Promoters. Mol. Cell.

[174] Javadekar S.M., Nilavar N.M., Paranjape A., Das K., Raghavan S.C. (2020). Characterization of G-quadruplex antibody reveals differential specificity for G4 DNA forms. DNA Res..

[175] Lafer E.M., Sousa R., Rich A. (1985). Anti-Z-DNA antibody binding can stabilize Z-DNA in relaxed and linear plasmids under physiological conditions. EMBO J..

[176] Boissieras J., Bonnet H., Susanto M.F., Gomez D., Defrancq E., Granzhan A., Dejeu J. (2024). IMab antibody binds single-stranded cytosine-rich sequences and unfolds DNA i-motifs. Nucleic Acids Res..

[177] Smolka J.A., Sanz L.A., Hartono S.R., Chédin F. (2021). Recognition of RNA by the S9.6 antibody creates pervasive artifacts when imaging RNA:DNA hybrids. J. Cell Biol..

[178] Hui W.W.I., Simeone A., Zyner K.G., Tannahill D., Balasubramanian S. (2021). Single-cell mapping of DNA G-quadruplex structures in human cancer cells. Sci. Rep..

[179] Martin R.M., de Almeida M.R., Gameiro E., de Almeida S.F. (2023). Live-cell imaging unveils distinct R-loop populations with heterogeneous dynamics. Nucleic Acids Res..

[180] Galli S., Melidis L., Flynn S.M., Varshney D., Simeone A., Spiegel J., Madden S.K., Tannahill D., Balasubramanian S. (2022). DNA G-Quadruplex Recognition In Vitro and in Live Cells by a Structure-Specific Nanobody. J. Am. Chem. Soc..

[181] Di Antonio M., Ponjavic A., Radzevičius A., Ranasinghe R.T., Catalano M., Zhang X., Shen J., Needham L.M., Lee S.F., Klenerman D. (2020). Single-molecule visualization of DNA G-quadruplex formation in live cells. Nat. Chem..

[182] Víšková P., Ištvánková E., Ryneš J., Džatko Š., Loja T., Živković M.L., Rigo R., El-Khoury R., Serrano-Chacón I., Damha M.J. (2024). In-cell NMR suggests that DNA i-motif levels are strongly depleted in living human cells. Nat. Commun..

